# Linking microbiome and stress hormone responses in wild tropical treefrogs across continuous and fragmented forests

**DOI:** 10.1038/s42003-023-05600-9

**Published:** 2023-12-12

**Authors:** Wesley J. Neely, Renato A. Martins, Camila M. Mendonça da Silva, Tainá Ferreira da Silva, Lucas E. Fleck, Ross D. Whetstone, Douglas C. Woodhams, W. Harrison Cook, Paula R. Prist, Victor H. Valiati, Sasha E. Greenspan, Alexandro M. Tozetti, Ryan L. Earley, C. Guilherme Becker

**Affiliations:** 1 Department of Biology, The University of Alabama, Tuscaloosa, AL 35487 USA; 2 Department of Biology, Texas State University, San Marcos, TX 78666 USA; 3 Department of Biology, and Center for Infectious Disease Dynamics, One Health Microbiome Center, The Huck Institutes of the Life Sciences, The Pennsylvania State University, University Park, PA 16802 USA; 4 Programa de Pos‑Graduacão em Biologia, Universidade do Vale do Rio dos Sinos, São Leopoldo, RS 93022‑750 Brazil; 5 Department of Biology, University of Massachusetts Boston, Boston, MA 02125 USA; 6 EcoHealth Alliance, 520 Eight Avenue, Suite 1200, New York, NY 10018 USA

**Keywords:** Microbial ecology, Microbiome

## Abstract

The amphibian skin microbiome is an important component of anti-pathogen defense, but the impact of environmental change on the link between microbiome composition and host stress remains unclear. In this study, we used radiotelemetry and host translocation to track microbiome composition and function, pathogen infection, and host stress over time across natural movement paths for the forest-associated treefrog, *Boana faber*. We found a negative correlation between cortisol levels and putative microbiome function for frogs translocated to forest fragments, indicating strong integration of host stress response and anti-pathogen potential of the microbiome. Additionally, we observed a capacity for resilience (resistance to structural change and functional loss) in the amphibian skin microbiome, with maintenance of putative pathogen-inhibitory function despite major temporal shifts in microbiome composition. Although microbiome community composition did not return to baseline during the study period, the rate of microbiome change indicated that forest fragmentation had more pronounced effects on microbiome composition than translocation alone. Our findings reveal associations between stress hormones and host microbiome defenses, with implications for resilience of amphibians and their associated microbes facing accelerated tropical deforestation.

## Abstract (web summary)

Short-term tracking of treefrogs in southern Brazil, alongside microbiome and stress hormone profiling, suggests that forest fragmentation impacts microbiome diversity and composition, and that these changes might be mediated by the host stress response.

## Introduction

Environmental stressors negatively impact wildlife through many different mechanisms. One such mechanism includes synergistic interactions between stress responses and immune function, which can influence host fitness and disease susceptibility under environmental stress^1–4^. Amphibians are particularly susceptible to environmental stressors, are among the most threatened taxa globally^5^, and have suffered major declines in recent years due to habitat loss and disease^6–10^. Habitat loss can concentrate populations in smaller areas, increase energy expenditure during movement, and increase host stress leading to hampered immune function^11–14^. Therefore, determining the crucial drivers linking habitat loss, fragmentation, and host fitness to diseases in wild amphibian populations is becoming an increasingly important area of research focus.

Glucocorticoid hormones, including cortisol and corticosterone, are important components of vertebrate immune function affecting host fitness^15–17^. These hormones, activated through the hypothalamus-pituitary-adrenal/interrenal (HPA/I) axis of the endocrine system, are central to host physiology, playing key roles in stress response, metabolism, cognition, and behavior^18–22^. Amphibians exhibit elevated glucocorticoid levels when experiencing environmental stressors^7,23–25^, and this adaptive response is crucial for survival because it enables animals to mount efficient responses to environmental threats^23,26^. However, stress responses become maladaptive over longer periods, as they can deplete energy stores and overtax organ systems^27,28^. The glucocorticoid-fitness (CORT-fitness) hypothesis was proposed to accommodate predicted relationships between glucocorticoid hormone levels and host fitness^15,29^. Under this hypothesis, environmental stressors that increase glucocorticoid levels should in turn decrease host fitness as energy demands increase^26,29^.

Another contributor to immune function is the host-associated microbiome, which, combined with the innate immune system, provides a defensive mechanism against pathogen colonization and growth^30–33^. In line with work on the microbiomes of other organisms, classifying protective aspects of the amphibian microbiome is complex and often conflicting between host species and study systems^34–36^. However, a growing number of bacteria with pathogen-inhibiting functions are being identified through in-vitro challenge assays^37–40^, which may provide a potential proxy for bacterial function in vivo. These include bacteria isolated from amphibian skin capable of inhibiting colonization and growth of the amphibian chytrid fungus, *Batrachochytrium dendrobatidis* (*Bd*)^40–43^, a globally distributed pathogen of great conservation concern for amphibians^8,44,45^. The outcome of pathogen infection may, therefore, be determined by adaptive shifts in the microbiome through enrichment with key anti-pathogen members, bolstering immune defenses^33,41,43,46–48^.

Host microbiomes can influence glucocorticoid levels through regulation of the HPA/I axis^49,50^, with induced increases in host glucocorticoid levels being linked with reductions in host microbiome community diversity and shifts in microbial composition^2–4^. While environmental disturbances also affect glucocorticoid levels^51^ and microbiome dynamics^52,53^, little research to date has addressed how these defensive mechanisms are affected by accelerated habitat loss and fragmentation. Thus, studying the links between microbiome function, hormonal responses, and host fitness is necessary to fully grasp the impact of anthropogenic habitat fragmentation on wildlife.

Ecological resilience refers to the ability of a community to resist or bounce back from disturbances without permanent and drastic changes to community structure or function^54,55^. Tropical amphibians move between aquatic, terrestrial, and arboreal habitats over short time periods^11^, all providing different microbial reservoirs for microbiome recruitment^56–58^. However, little is known about how host movement patterns and skin microbiome dynamics change over time in response to habitat fragmentation, a major source of ecological disturbance. On one hand, hosts moving seasonally through drastically different habitats could result in regular changes to the structure of their microbiomes^59^, priming these communities for resilience in response to disturbances caused by habitat fragmentation. On the other hand, habitat fragmentation could inflict stress on hosts beyond that incurred through natural movement paths, impacting resilience of amphibian communities by disrupting dispersal^60–62^. This would in turn alter small-scale host movement behavior^63,64^ and associated microbial recruitment. Because changes in the microbiome can be associated with increased disease risk^65–67^, habitat fragmentation impacting both microbiome community stability through disruptions to host movement could increase disease risk.

To study the relationship between microbiome composition and anti-pathogen function, glucocorticoid levels, body condition, and *Bd* infection in the context of habitat fragmentation, we conducted a longitudinal study tracking a cohort of translocated and non-translocated frogs. Our focal species, the forest-associated blacksmith treefrog, *Boana faber*, is a large-bodied tropical tree-frog that moves through aquatic, arboreal, and terrestrial habitats over relatively short timeframes^68,69^. We hypothesized that habitat fragmentation would negatively impact hosts through reductions in suitable habitat and restrictions to dispersal. We predicted that translocating frogs from continuous to fragmented forests would result in lower body condition, higher levels of glucocorticoid hormones, declines in putative anti-pathogen function, and increases in pathogen infection when compared to frogs translocated to sites within continuous forest. Our findings uncover connections between stress hormones and microbiome defenses, with potential implications for the resilience of microbiomes in the face of habitat fragmentation.

## Results

### Field results

**Fig. 1:**
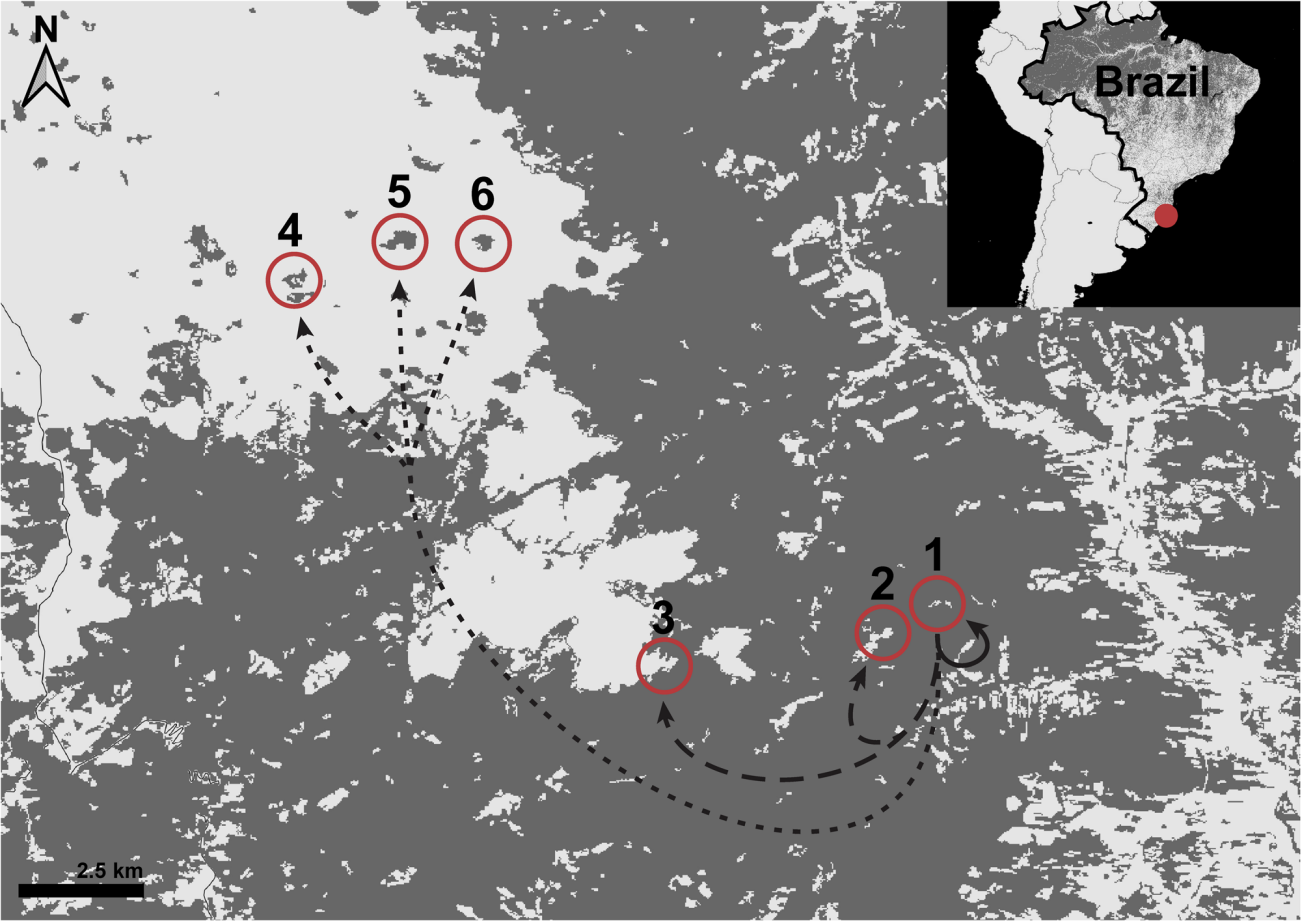
Map of sampling locations. Forested areas are shown in dark gray and non-forested areas are shown in light gray. Treatments are labeled as follows: Continuous-Control (1), Continuous-Translocated (2–3), and Fragment-Translocated (4–6). Arrows show direction of translocation for each treatment, with all frogs originating at site 1.

We recaptured *B. faber* across habitats including emergent plants in water bodies, leaf litter, tree branches, and bromeliad tanks (Supplementary Fig. 1; Supplementary Table 1). Individuals at continuous forest sites were primarily found on the ground (24%) while those at fragments were primarily found in bromeliads (18%; Supplementary Fig. 1; Supplementary Table 1). We recorded nine predation events during our study. At one site (#3, Fig. 1), four out of five frogs were predated upon during a single inter-sampling period, and one additional frog was predated upon two weeks later. We recovered the four transmitters 900 m to the East of site #3, covered in bite marks and scattered among trails in tall grass. These trails were also marked with Crab-eating Fox feces. Three transmitters were recovered at site #1 (Fig. 1) with similar signs of predation. One transmitter was found eaten by a false fer-de-lance (*Xenodon neuwiedii*) at site #2 (Fig. 1). Cumulatively, 23% of radio-tagged frogs were predated upon to the best of our knowledge.

### Bd infection dynamics

We found 15% *Bd* prevalence across the entire sampling period (*n* = 112) and for the initial sampling alone (*n* = 40). We detected 11% *Bd* prevalence at the second sampling, 21% at the third sampling, 18% at the fourth sampling, and 14% at the fifth sampling. Pathogen loads ranged from 0 to 3,707 ITS gene copies, with a mean of 108 ± 464 gene copies. *Bd* infection load values were similar between treatments for the sequential sampling periods (Continuous-Control: 84 ± 247 gene copies; Continuous-Translocated: 158 ± 588 gene copies; Fragment-Translocated: 201 ± 719 gene copies). During our sampling, we detected 8 frogs that gained *Bd* infection, 6 frogs that cleared *Bd* infection, and 4 frogs that gained then subsequently cleared infection (Supplementary Fig. 1). It is important to note that low-load infection can be difficult to detect so some instances of gaining or clearing of infection may be attributed to undetected low loads.

### Microbiome spatiotemporal dynamics

After all filtering steps we recorded 504 bacterial sOTUs across all samples with the dominant families (based on number of sequence reads) being Proteobacteria and Bacteroidetes. A shift occurred in the microbiome after the first two sampling periods (~10 days) across all treatments, where the dominant phylum of bacteria shifted from Proteobacteria (driven by two sOTUs of *Pseudomonas*) to Bacteroidetes (driven by one sOTU of *Chryseobacterium*; Supplementary Fig. 2). All three of these sOTUs were identified as *Bd*-inhibitory, matching sequences of our cultured isolates. Using linear discriminant analysis effect size analysis to identify differentially abundant sOTUs between samples, we found 85 sOTUs higher in abundance in frogs pre-translocation, 8 higher in frogs post-translocation to the site of capture (Continuous-Control), 7 higher in frogs translocated to continuous forest sites (Continuous-Translocated), and 19 sOTUs higher in frogs translocated to forest fragments (Fragment-Translocated; Supplementary Fig. 3, Supplementary data 1). Additionally, three of our *Bd*-inhibitory isolates were detected as differentially abundant, two pre-translocation across all treatments (*Pseudomonas fragi* and *Stenotrophomonas*), and one post-translocation across all treatments (Enterobacteriaceae; Supplementary Fig. 3, Supplementary data 1).

**Fig. 2:**
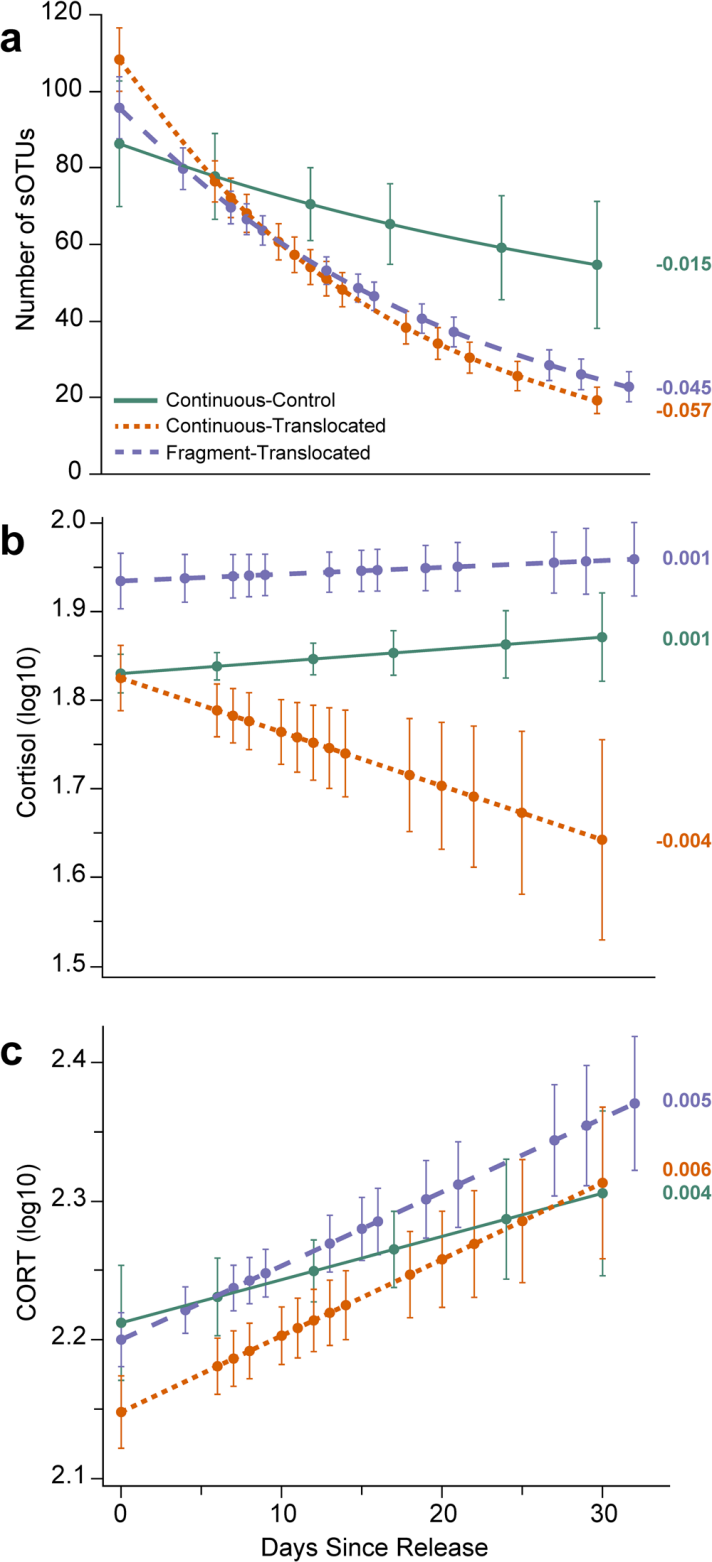
Changes in microbiome diversity and glucocorticoid levels over time between the three treatments. Covariations show bacterial sOTU richness (**a**), cortisol levels (**b**), corticosterone (CORT) levels (**c**) and number of days since release, split by treatment group: Continuous-Control (green), Continuous-Translocated (orange), and Fragment-Translocated (purple). Relationships are based on bootstrap aggregated residuals saved from generalized linear models. Error bars show bootstrap aggregated standard deviations of the predictors. Slope for each line is shown.

We found significant effects of days since release (*F*_[1,93.52]_ = 47.24, *P* < 0.0001) and the interaction between treatment and days since release (*F*_[2,92.54]_ = 4.61, *P* = 0.0123; Fig. 2a) on sOTUs richness. The Continuous-Control treatment had a significantly different slope than the translocated treatments (Diff = 0.023 ± 0.010 [95% CI: 0.001, 0.045]), with no difference between Continuous-Translocated (Diff = −0.019 ± 0.012 [95% CI: −0.047, 0.008]) or Fragment-Translocated treatments (Diff = −0.007 ± 0.008 [95% CI: −0.025, 0.012]; Fig. 2a).

Transmitter attachment showed no evidence of an effect on sOTU richness (*F*_[3,28]_ = 0.49, *P* = 0.692) or proportion of *Bd*-inhibitory bacteria (*F*_[3,28]_ = 0.359, *P* = 0.783).

### Glucocorticoid dynamics and host body condition

Results on the relationships between glucocorticoids and body condition between treatments are being published separately. We found significant variation in cortisol levels between treatments (*F*_[2,28.28]_ = 6.54, *P* = 0.005; Fig. 2b) with higher cortisol levels for the Fragment-Translocated group. However, log cortisol levels were non-significantly higher in the Fragment-Translocated group (1.92 ± 0.10) than the Continuous-Control and Continuous-Translocated groups initially (1.84 ± 0.09; 1.84 ± 0.16, respectively), so this result may be partially biased. There was no relationship between days since release and cortisol (*F*_[1,92.44]_ = 0.06, *P* = 0.808; Fig. 2b), and no significant differences in the slope of that relationship between treatments (Continuous-Control: Diff = 0.001 ± 0.003 [95% CI: −0.005, 0.008]; Continuous-Translocated: Diff = −0.004 ± 0.003 [95% CI: −0.011, 0.003]; Fragment-Translocated treatments: Diff = 0.001 ± 0.002 [95% CI: −0.004, 0.005]), although there was a nonsignificant negative trend in cortisol levels over time for the Continuous-Translocated treatment (Fig. 2b).

There was no significant variation in corticosterone levels between treatments (*F*_[2,15.2]_ = 2.34, *P* = 0.130; Fig. 2c) but, we found a significant relationship between days since release and corticosterone levels (*F*_[1,103.2]_ = 16.15, *P* < 0.001) where corticosterone increased linearly over time. There were no significant differences in the slope of that relationship among treatments (Continuous-Control: Diff = −0.001 ± 0.002 [95% CI: −0.007, 0.005]; Continuous-Translocated: Diff = 0.001 ± 0.002 [95% CI: −0.005, 0.006]; Fragment-Translocated treatments: Diff = 0.000 ± 0.002 [95% CI: −0.004, 0.004]; Fig. 2c).

Body condition did not differ significantly among treatments (*F*_[2,35.04]_ = 2.59, *P* = 0.089), but had a decreasing trend from Continuous-Control (β estimate = 0.72 ± 0.03) to Continuous-Translocated (β estimate = 0.68 ± 0.02) to Fragment-Translocated treatments (β estimate = 0.65 ± 0.02).

### Community trajectory analysis

**Fig. 3:**
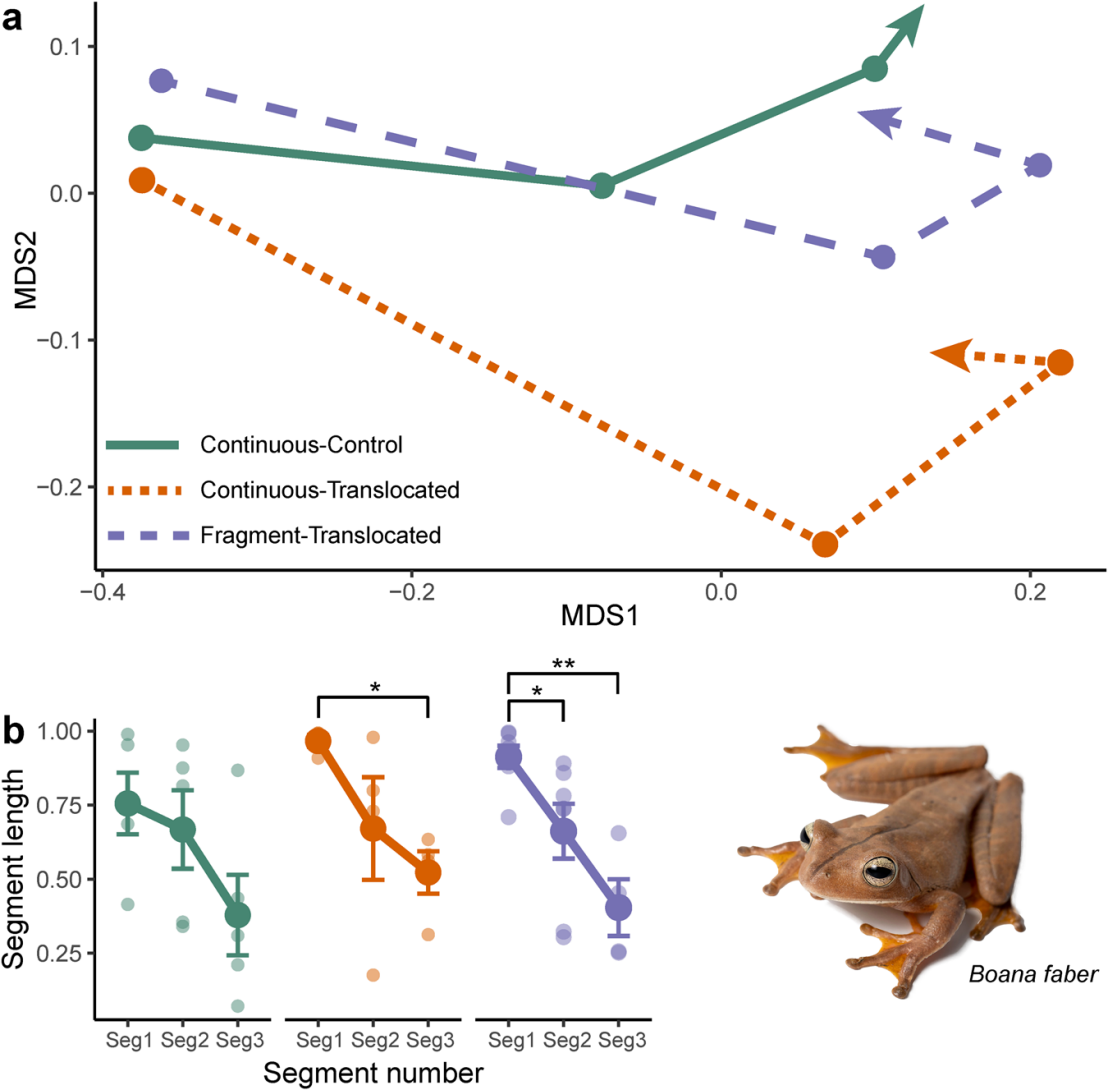
Shifts in microbiome community composition over time between treatments. Plots showing (**a**) shifts in microbiome composition trajectories over time, with centroids averaged for each timepoint within each treatment and (**b**) average segment lengths with standard error bars for each treatment. Brackets and asterisks (**b**) refer to difference based on *post hoc* testing (*p < 0.05, **p < 0.01). Connecting lines (**b**) visually highlight changes in segment length and do not represent linear regression fit. The three treatments are Continuous-Control (solid green line, *n* = 5), Continuous-Translocated (short dashed orange line, *n* = 4), and Fragment-Translocated (long dashed purple line, *n* = 9). Ordination is based on non-metric multidimensional scaling with Bray–Curtis distances.

We found similar patterns of multidimensional movement for microbial community composition across the three treatments through CTA (Fig. 3a, Supplementary Fig. 4). All three treatments had intermediate values for directionality (0.405–0.433), indicating similar net changes in multidimensional direction. We found no significant differences in overall directionality (*F*_[2,12]_ = 0.59, *R*^2^ = 0.09, *P* = 0.568) or mean angles (*F*_[2,10]_ = 0.62, *R*^2^ = 0.11, *P* = 0.556) of trajectories between treatment groups (Supplementary Table 2). We did, however, find a significant reduction in segment length over time in the Continuous-Translocated (*F*_[2,9]_ = 4.31, *R*^2^ = 0.49, *P* = 0.049) and Fragment-Translocated (*F*_[2,13]_ = 10.30, *R*^2^ = 0.61, *P* = 0.002) treatments, but no change for frogs in the Continuous-Control treatment (*F*_[2,12]_ = 2.49, *R*^2^ = 0.29, *P* = 0.125; Fig. 3b). This metric indicates the magnitude of change in microbiome composition, with longer segment lengths indicating greater change in the microbiome. *Post hoc* tests revealed that for the Continuous-Translocated treatment, only the first and third segment lengths differed (Diff = 0.44 ± 0.15, *P* = 0.0433). For the Fragment-Translocated treatment, the first segment length differed from the second (Diff = 0.30 ± 0.10, *P* = 0.0228) and third (Diff = 0.46 ± 0.11, *P* = 0.0020).

**Fig. 4:**
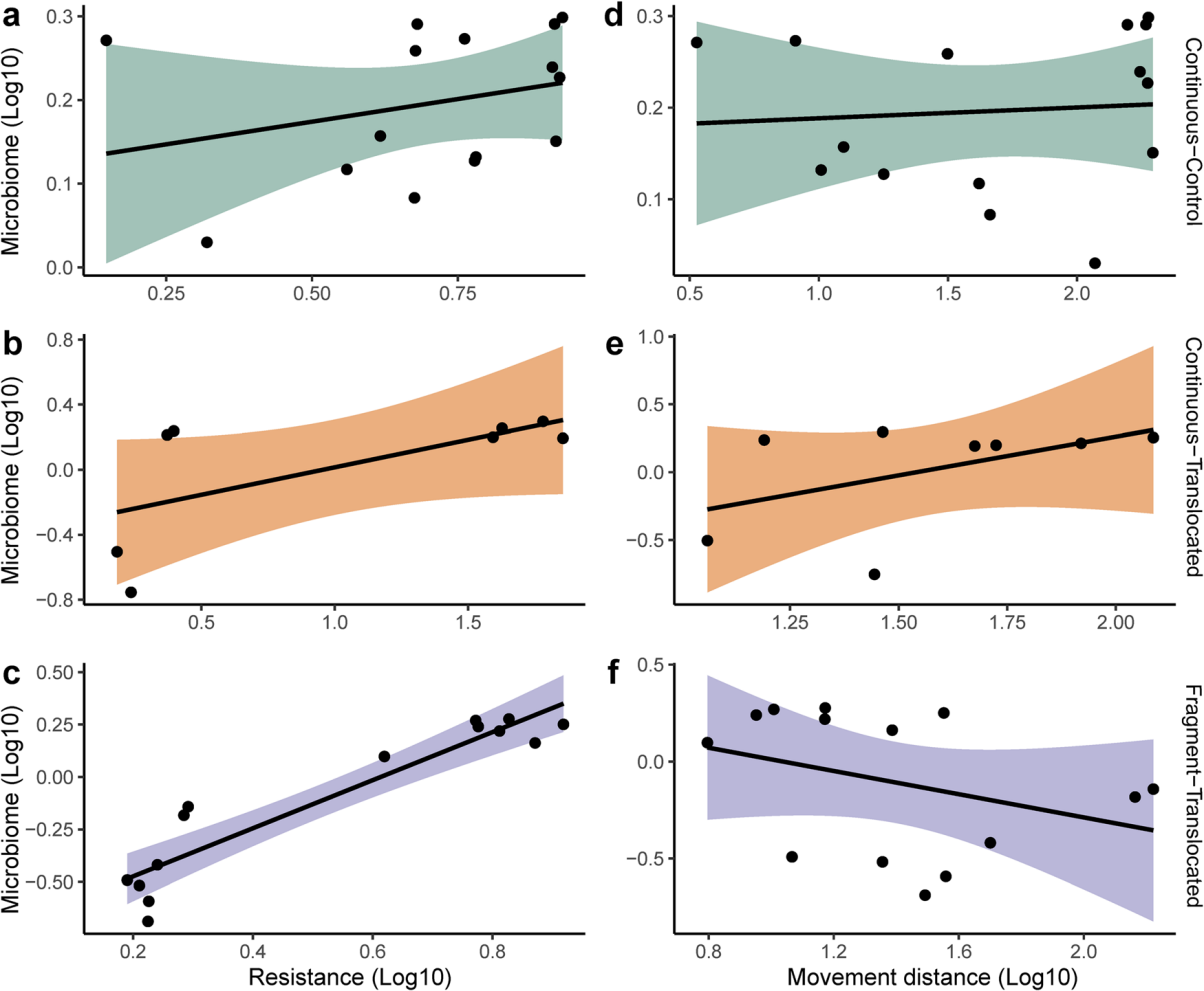
Correlations between shifts in microbiome compositional and host movement paths. Scatterplots show microbiome segment length correlations with resistance to movement (**a**–**c**) and movement distance (**d**–**f**) for the three treatments: Continuous-Control (**a**, **d**; green), Continuous-Translocated (**b**, **e**; orange), and Fragment-Translocated (**c**, **f**; purple). Shaded regions around lines show 95% confidence of fit.

Average movement distance was 92 ± 81 m for Continuous-Control frogs, 49 ± 38 m for Continuous-Translocated frogs, and 41 ± 50 for Fragment translocated frogs. Average resistance to movement was 4.67 ± 2.32 for Continuous-Control frogs, 26.75 ± 29.29 m for Continuous-Translocated frogs, and 3.08 ± 2.59 for Fragment translocated frogs. Using GLM on log-transformed variables, we found a significant relationship between host movement paths and microbiome segment length in the Fragment-Translocated treatment (whole model test: *F*_[2,11]_ = 41.36, *R*^2^ = 0.88, *P* < 0.001; Fig. 4c). Specifically, there was a strong positive relationship between microbiome segment length and habitat resistance (β = 1.20 ± 0.14, *P* < 0.001), but not host movement distance (β = 0.09 ± 0.10, *P* = 0.395). Conversely, we found no relationship between host movement paths and microbiome segment length in Continuous-Control (*F*_[2,12]_ = 0.59, *R*^2^ = 0.09, *P* = 0.571) or Continuous-Translocated treatments (*F*_[2,5]_ = 2.00, *R*^2^ = 0.44, *P* = 0.230; Fig. 4).

### Structural equation models

**Fig. 5:**
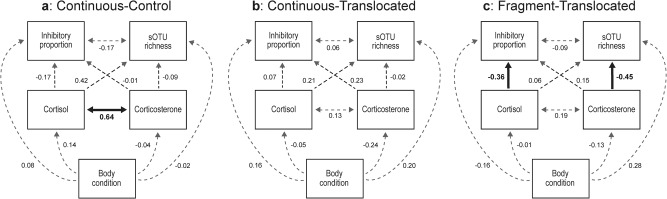
Path model diagrams showing direct and indirect connections between variables, split by treatment. Path models show relationships between variables for Continuous-Control (**a**), Continuous-Translocated (**b**), and Fragment-Translocated (**c**) treatment groups. Double headed arrows indicate correlations. Statistically significant (*p* < 0.05) paths are shown with solid bolded lines and non-significant paths with dashed gray lines. Standardized estimates are shown next to all paths.

Through structural equation modeling, we found relationships between glucocorticoids and the microbiome for frogs in the Fragment-Translocated treatment (Fig. 5c; Supplementary data 2). Specifically, higher cortisol levels were associated with a lower proportion of *Bd*-inhibitory bacteria (β = −0.35 ± 0.15, *P* = 0.032), and higher corticosterone levels were associated with lower sOTU richness (β = −0.45 ± 0.15, *P* = 0.007). None of these strong relationships were evident in Continuous-Control or Continuous-Translocated (Fig. 5a, b). Additionally, we found a strong positive correlation between cortisol and corticosterone levels (β = 0.63, *P* < 0.001), but only for non-translocated frogs (Fig. 5a; Supplementary data 2)

### Repeatability analysis

We detected high repeatability, or relatively low within-individual variation, for proportion of *Bd*-inhibitory bacteria in the microbiome in both the Continuous-Control (0.66 [95% CI: 0.28, 1.07]) and Fragment-Translocated treatments (0.43 [95% CI: 0.12, 0.77]), but not the Continuous-Translocated treatment (0.09 [95% CI: −0.09, 0.27]). We found low repeatability for sOTU richness in the Continuous-Control (0.11 [95% CI: −0.01, 0.25]), Continuous-Translocated (0.03 [95% CI: −0.10, 0.15]), and Fragment-Translocated treatments (0.01 [95% CI: −0.03, 0.05]).

## Discussion

In this study, we showed that translocation and habitat fragmentation influence the host-associated microbiome and glucocorticoid dynamics. Through a combination of bacterial diversity loss and community restructuring, our results indicate a state shift in microbiome community equilibrium, potentially driven by intrinsic stress-associated factors influencing antimicrobial production^25^ or extrinsic factors associated with environmental reservoirs of bacteria^58,70,71^. It is plausible that our results showing convergence in microbiome similarity among frogs stems from stress responses in the host, causing a decrease in more transient microbial taxa. This hypothesis is further supported by the following findings: (1) elevated cortisol in frogs translocated to forest fragments compared to those translocated to continuous forest, (2) decreased number of differentially abundant taxa after translocation, (3) contrasting shifts in community composition in the microbiome of frogs translocated to forest fragments compared to other treatments, and (4) higher microbiome dissimilarity with greater resistance to host movement for frogs translocated to fragments. Together, our findings indicate a strong link between amphibian stress response and microbial community composition, and may indicate reductions in microbiome community resilience when amphibians are faced with external stressors like habitat fragmentation^55,72–74^.

Resilient systems can be characterized by their robustness, which is their ability to resist changes in structure and maintain key functions after disturbance. Resilient systems also can be characterized by their recovery, which is their capacity to return to an equilibrium state and restore key functions after disturbance^55,73,75^. Biological communities can increase resilience through mechanisms like functional redundancy, where different members perform the same function, and functional diversity, or the breadth of functions that different members contribute^76^. The host-associated microbial community could therefore be viewed as a resilient system where functional diversity of certain bacterial groups resist or bounce back from perturbations such as pathogen invasion^77^. In our system, for instance, a resilient microbiome could be allowing for redundancy of *Bd*-inhibitory function across many host-associated bacterial taxa (e.g., *Pseudomonas* [Proteobacteria] and *Chryseobacterium* [Bacteroidetes]) despite observed turnover in this system over time. One recent study found temporal stability of enzyme production in tropical forest-associated soil microbiomes despite significant temporal turnover in microbial composition^78^, which indicates resilience through functional diversity^73,75^. Amphibian skin microbial communities are recruited directly from environmentally available pools of microbes^56,58,79^ therefore, despite shifts in the available taxa for recruitment, the same functional groups often persist.

By tracking shifts in skin microbiome composition across space and time, we found relatively similar patterns in directional change among our three treatments between sampling periods. Overall trajectory directionality values indicate that microbiome composition in all treatments may be stabilizing and slowly returning to the initial pre-disturbance state (Fig. 3a)^80,81^. Average microbiome composition trajectories across all three treatments had large initial shifts after tracker attachment and continued to move in similar directions over time. However, only translocated frogs had significant reductions in segment length over time, and only frogs translocated to fragments had a significantly reduced shift in microbiome composition after the first pair of samples. Further, only the treatment translocated to fragments had associations between habitat resistance to host movement and the magnitude of microbiome change. While in general frogs at fragmented habitats had smaller movement distances and tended to stay within the forest edge, this strong positive relationship indicates that even small movements across more open habitats is enough to drive change in microbial community composition. This further supports stress-associated drivers of microbiome compositional change across space and time.

It was not possible to capture microbiome recovery (return to initial states) within the timeframe of our study due to natural seasonal variation in the microbiome^59,82^. Based on changes in trajectory path lengths, however, we see that microbiome composition for frogs at continuous forest sites continued to have larger shifts after the first pair of samples than those translocated to forest fragments. It is possible, therefore, that microbiome communities of frogs at continuous forest sites may return to a community structure matching initial states after more time has passed, whereas the microbiome community structure for frogs at forest fragments may reach a new stable equilibrium. We found that skin microbiomes were dynamic across the study period, regardless of treatment, which could be driven by the drastic change in rainfall during this period (Supplementary Fig. 5)^83^. However, we also found consistent decreases in sOTU richness and increases in corticosterone levels over time across all three treatments. Therefore, chronic stress from repeated handling and radio-tag attachment may be contributing to some of the dynamics that we report. We documented a pronounced shift in the dominant microbial taxa from Proteobacteria of the genus *Pseudomonas* to Bacteroidetes of the genus *Chryseobacterium* during our study (Supplementary Fig. 2). Interestingly, these two taxa were also detected as abundant *Bd*-inhibitory bacterial isolates in our database. Additionally, the bacteria *Janthinobacterium lividum* (Proteobacteria), which has known anti-fungal properties^84–86^, was detected as more abundant post-translocation. Our findings, both observationally and through differential abundance analysis, showing maintenance of *Bd*-inhibitory properties despite turnover in taxonomic composition, further support a potentially resilient (i.e., robust) microbiome in this study system.

**Fig. 6:**
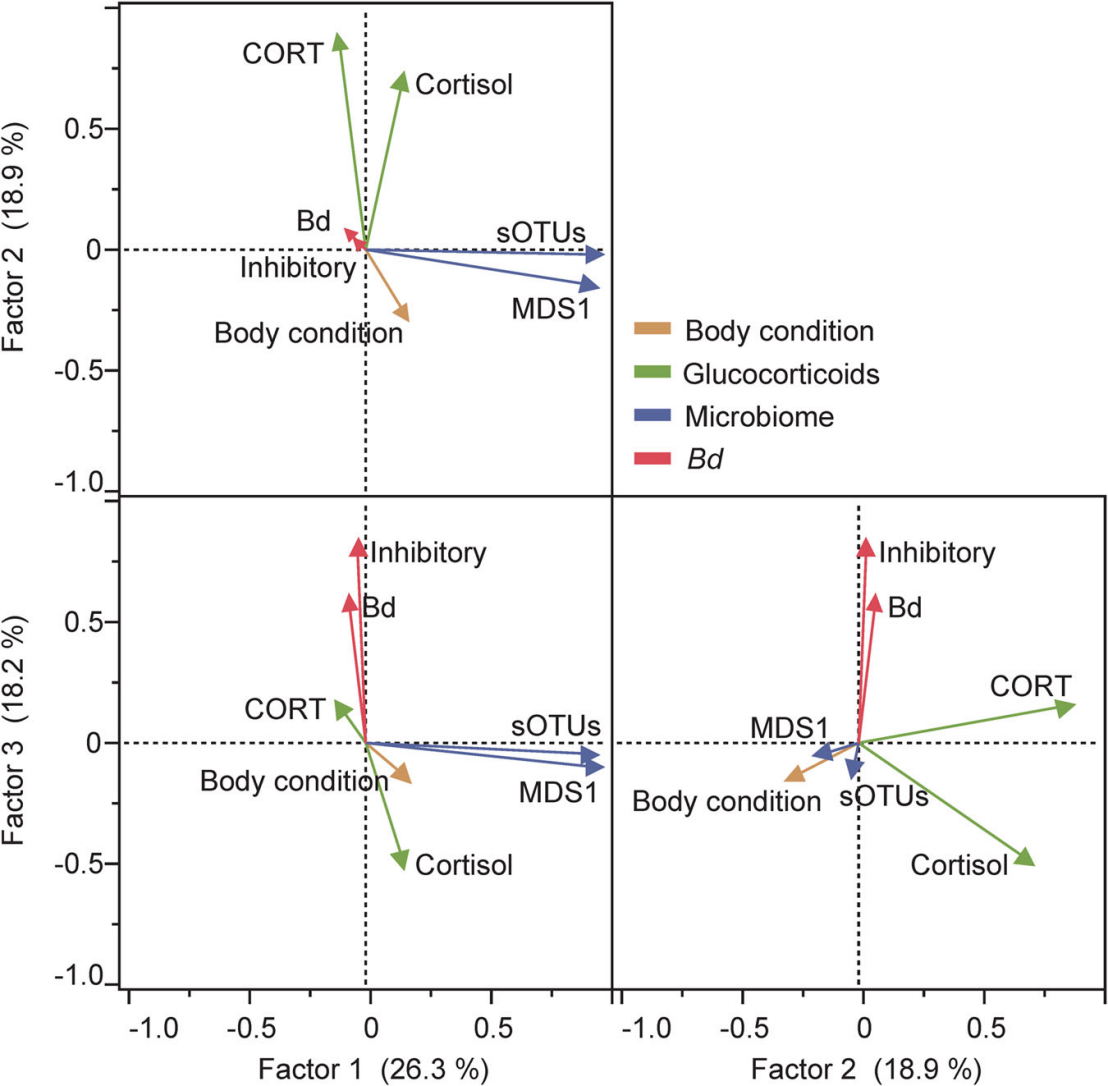
Factor loading bi-plot based on principle component analysis. Bi-plot includes *Bd* infection loads, body condition (mass/SVL), cortisol, corticosterone (CORT), sOTU richness, microbiome similarity (MDS1), and inhibitory proportion of bacteria. Colors represent the four major classifications that loaded together. Loading values can be found in Supplementary Table 4.

Putative skin microbiome function and diversity were linked to another important component of amphibian immune response: mucosal glucocorticoid concentrations^25,87^. This relationship was detected only for frogs translocated to forest fragments. Habitat fragments could limit amphibian dispersal and drive movement into less suitable habitats, especially for large-bodied forest dwelling tree frogs like *B. faber*. Stress incurred through disrupted locomotion may be concurrently increasing glucocorticoid levels while movement across less suitable habitats leads to reductions in microbiome diversity and putative function. We also found a high level of repeatability for the proportion of *Bd*-inhibitory bacteria in the microbiome, meaning that there were consistent differences among individuals over time in proportions of detected *Bd*-inhibitory bacteria. For example, differences between frogs that had high or low initial proportions of *Bd*-inhibitory bacteria were maintained throughout the sampling period, regardless of shifts in microbiome species composition. This could indicate that this putative metric of microbiome function may be intrinsic to each frog and less dependent on environmental conditions^88^. This is supported by the fact that all frogs were collected from the same site and likely have low genetic variability between individuals. Further, the lack of repeatability in bacterial richness and lack of correlation between bacterial richness and proportion of *Bd*-inhibitory bacteria in the microbiome strongly indicate that the proportion of *Bd*-inhibitory bacteria is not simply a function of microbiome diversity. Additionally, the co-variation of *Bd* with *Bd*-inhibitory bacteria (Fig. 6) may indicate an adaptive microbiome response^33^, perhaps facilitated by glucocorticoids (Fig. 6). Frogs translocated to forest fragments experienced greater stress, as they were moved farthest and to less suitable habitats. Correlations between glucocorticoid levels and microbial diversity and function, consistent patterns of body condition and glucocorticoid levels between frogs, and higher cortisol levels than the Continuous-Translocated treatment could reflect individualized physiological stress responses to the Fragment-Translocated treatment. Of important consideration, in-vitro inhibition of *Bd* does not necessarily indicate in-vivo bacterial function, so these results indicating associations with this metric require experimental validation to determine true anti-pathogen function.

The relationship between glucocorticoids and the microbiome has received relatively little research attention, but some studies have reported general reductions in host-associated microbial diversity with increased glucocorticoid levels^2–4,89–91^. However, other studies report higher microbial diversity^1^ or increases in potential opportunistic pathogenic taxa with increasing glucocorticoid levels^4,90^. Interestingly, two studies report that cortisol levels were negatively correlated with abundance of potentially probiotic lactic acid bacteria^4,89^. While mechanisms driving the negative relationship between cortisol and inhibitory bacteria in our study are unknown, some potential routes include direct toxicity of cortisol on bacteria capable of producing anti-fungal metabolites, or host metabolic impairment altering mucosal conditions needed to promote beneficial bacterial growth^92,93^. Similar mechanisms could be driving the negative relationship between corticosterone and microbial richness that we revealed in this study. We also found a correlation between cortisol and corticosterone in the Continuous-Control treatment, but not in the two translocated treatments. This could indicate that the function of these hormones is context-dependent, diverging in more challenging environments. It is important to note that we did not collect data on the maximal glucocorticoid response of these frogs, so we cannot state whether higher glucocorticoid levels recorded in this study are indicators of stress, or if they fall within the natural level of variation in these hormones. Our results contribute to the growing body of literature connecting glucocorticoid production with microbiome function by demonstrating a correlation between mucosal cortisol levels and bacterial inhibition of the amphibian fungal pathogen *Bd*.

Through our sampling design tracking individual frogs across natural movement paths, we were able to document changes in *Bd* infection for our focal amphibian species. More discussion on habitat selection and predation can be found in the supplemental materials (Supplementary note 1). Prevalence was consistent across our study, around 15% of individuals infected, and did not undergo significant shifts after translocation to forest fragments as predicted. During the tracking period, multiple individuals moved between infected and uninfected states. Frogs are regularly exposed to *Bd* during their natural movements through water bodies, bromeliad reservoirs, or contact with other frogs^94–96^, but our findings demonstrate a temporary and seemingly non-lethal enzootic nature of *Bd* infection in this system. Our study area lies at around 1000 meters of elevation and falls within the southernmost extent of the Brazilian Atlantic Forest. This area is characterized by consistent cool temperatures (15–20 °C; Supplementary Fig. 5) and high humidity (75–85%; Supplementary Fig. 5), conditions that are considered to be favorable for the growth and transmission of *Bd*^97,98^. In 2014, Becker et al.^53^ conducted field work studying a different species of treefrog from the same area as our study and found *Bd* prevalence at around 60%, adding evidence of a suitable environment for *Bd* proliferation. We found comparatively lower *Bd* prevalence and infection loads in our study, possibly indicating *Bd* resistance resulting from previous epizootic declines with subsequent population recovery^44,99,100^. Various mechanisms could be driving the apparent resistance to *Bd* infection in this system including immune-associated genetic diversity of the major histocompatibility complex^101^ and production of antimicrobial peptides^102^. However, coupled with our findings of high proportions of *Bd* inhibitory bacteria, it is possible that protective anti-fungal members in the microbiome are limiting *Bd* colonization and growth.

In conclusion, we found correlations between increased host stress, reduced microbiome function, and altered microbiome trajectories, likely exacerbated by habitat fragmentation. We report associations between the skin microbiome and glucocorticoid hormone production in stressful environments, with potential implications for stress-associated influences on microbial recruitment or retention. Additionally, we reveal a potential capacity for resilience in our study system, with maintenance of putative *Bd*-inhibitory function despite major shifts in microbiome composition. Return of microbiome community composition to initial states was not completed over the study timeframe, and we found indications that forest fragmentation may have a greater negative impact on microbiome composition than the stress of translocation alone based on the rate of microbiome change and the observed strong association with habitat resistance. Combined, our results reveal connections between amphibian skin defenses and innate stress response, with implications for amphibian stability in the face of accelerated forest fragmentation in the tropics.

## Methods

### Field work

From December 2019 – January 2020, we tracked the skin microbial assemblage, glucocorticoid levels (cortisol and corticosterone), and *Bd* infection status of wild *B. faber* in southern Brazil. We captured 30 individual treefrogs (26 male and 4 female) from a single large breeding population located 1 km East of Pró-Mata field station in São Francisco de Paula, Rio Grande do Sul, Brazil (−29.4809, −50.1752) at the start of the breeding season in December. Capturing all frogs at a single site within continuous forest reduced any site-specific and population-specific differences in infection prevalence, skin microbial composition, and genetic factors at the beginning of the experiment. The municipality of São Francisco de Paula is characterized by mixed *Araucaria* deciduous forest and is flanked by large areas of grassland intermixed with forest patches and silviculture to the west^103^. Forest patches in this area are likely created by nucleation, or forest expansion, rather than fragmentation^104,105^. However, in the context of this study, these patches can act as forest fragments for frogs translocated from pristine, continuous habitats. During our study period, rainfall was lower in December and drastically increased in January, matching historical (prior 3 years) averages (Supplementary Fig. 5).

To monitor individual frogs, we attached nanotag VHF transmitters (Lotek) using microcapillary silicone tubes threaded with 28 gauge galvanized steel wire in a belt design, as recommended by Rowley and Alford^106^. The resulting transmitters were approximately 3 g in weight. We tagged each individual frog with coded nanotag transmitters (NTQB-6-1 - Lotek Wireless INC; operating frequency 140–175 Mhz) and monitored individual movement using a Biotracker VHF radio receiver and a 3-element folding Yagi antenna. Only frogs of adequate size (transmitter not to exceed 10% of body weight) were used, averaging 56.6 ± 8.5 g.

We split the 30 radio-tagged frogs into groups of 5 frogs that we released at 6 sites: 1 group at the site of initial capture (Continuous-Control treatment), 2 groups to other sites within the same continuous forest (Continuous-Translocated treatment) and 3 groups to sites in forest fragments (Fragment-Translocated treatment). Translocating frogs to forest fragments was meant to mimic the effects of sudden forest fragmentation common in areas facing intense logging pressure. These three treatments allowed us to compare the effects of translocation and habitat fragmentation while controlling for the effect of radio-transmitter attachment. All three forest fragments covered an area less than 0.3 square kilometers and were approximately 12 km from the original site of capture (Fig. 1). Sample sizes were chosen to allow for generation of averages within each site while minimizing the number of individuals to allow for more frequent monitoring.

After initial release, we recaptured frogs using radiotelemetry every ~5 days over 6 weeks (December 2019 – January 2020), with a maximum of 6 visits to each site. At initial capture and each recapture, we collected precise GPS coordinates, measured snout-vent length (SVL) and body mass, and collected skin swabs. We handled each frog with new gloves and rinsed frogs with distilled water prior to swabbing, taking care to remove any debris around the belt. Swabbing, using a sterile rayon swab (Medical Wire®), consisted of 10 strokes down the axilla and oblique of each frog and 5 strokes on the bottom of each foot^107^. We stored swabs on ice in 1.5 mL sterile screwcap tubes in the field, then at −20 °C until DNA extraction. We also collected an additional foam swab (PurSwab®) at each capture for stress hormone analysis^108^ that was stored in 1 mL of molecular-grade ethanol. At the end of the tracking period (January 2020), we swabbed frogs, removed radio tags, then released frogs at the location of last capture.

In total we recaptured and sampled radio-tagged frogs 112 times. Due to natural mortality (predation), we added 10 frogs (9 male and 1 female) after the start of the experiment by repurposing recovered radio tags. These tags were disinfected and checked for functionality before redeployment. We recaptured each frog an average of 3 times, with 28 frogs recaptured twice, 19 recaptured 3 times, 17 recaptured 4 times, 7 recaptured 5 times, and 2 recaptured 6 times. We opportunistically sampled resident (non-transmitter) conspecifics at the end of the tracking period at the site of original capture. Additionally, initial samples from the 10 frogs captured mid-study and redeployed with recovered transmitters are also used as resident samples, resulting in 17 resident samples.

All work on wild *B. faber* was conducted under appropriate permits (Instituto Chico Mendes – SISBIO #70883-1) and followed ethical regulations of institutional animal care and use committees from The University of Alabama (IACUC-UA #19-07-2547) and the Universidade do Vale do Rio dos Sinos (CEUA-UNISINOS #PPECEUA10.2019).

### Bacterial culturing and secondary metabolite collection

To obtain a putative metric of *Bd*-inhibitory potential of the microbiome of this host species, we used 44 swabs taken from *B. faber* in another Atlantic Forest site in the state of São Paulo, Brazil from 2020 to 2021. Using bacteria collected from the same species used in this study provides additional power to this metric of inhibition. These swabs were stored in 1 mL of sterile cryomedia (1% tryptone and 20% glycerol) and kept at −20 °C until culturing. We thawed and vortexed tubes containing cryomedia to evenly suspend bacteria before plating 20 µL on R2A agar. The inoculum was spread using sterile spreader bars to evenly distribute bacterial cells over the plate. Each plate was incubated at 21 °C for up to two weeks until colony growth plateaued. As they appeared, we picked unique colonies and re-streaked on fresh R2A agar to confirm purity prior to cryopreservation.

To collect bacterial secondary metabolites for use in *Bd* inhibition assays, 10 µL of cryopreserved culture was added to 3 mL of 1% tryptone broth in 5 mL borosilicate culture tubes. Cultures were incubated on a shaker for 72 h at room temperature to allow depletion of nutrients in the media. Bacterial suspensions were transferred to 2 mL microcentrifuge tubes and centrifuged at 7500 × *g* for 10 min to pellet bacteria. Cell-free supernatant was decanted and filtered through a 0.22 µm syringe filter prior to freezing at −20 °C for storage until inhibition assays commenced.

### Bd inhibition assays

Inhibition assays followed methods described by Siomko et al.^43^. Live *Bd* zoospores were inoculated on 1% tryptone agar plates and grown at 21 °C until zoosporangia matured. Upon sporangia rupture, plates were flooded with sterile 1% tryptone broth to collect fresh zoospores. Zoospore solution was then passed through sterile filter paper (10 µm porosity) to remove debris and sporangia. Zoospores were then quantified using a hemacytometer and diluted to 2 ×10^6^ zoospores/ml. In flat-bottom tissue culture-treated 96-well plates, 50 µL of zoospore solution was pipetted into positive control and experimental wells to yield a total zoospore count of 100,000 zoospores per well.

Three technical replicates for each control and experimental group were used. We added 50 µL sterile 1% tryptone broth to positive control wells and 50 µL sterile artificial pond water^109^ to nutrient-depleted controls to simulate lack of nutrients in experimental wells with bacterial cell-free supernatant. Experimental wells received 50 µL of bacterial cell-free supernatant. Heat-killed zoospores (incubated 1 h at 70 °C) were added to negative control wells with sterile 1% tryptone broth.

All wells were gently pipetted to evenly mix contents before plates were sealed with Parafilm and incubated at 21 °C. *Bd* growth was measured via optical density (OD_490_) on a spectrophotometer at days 0, 3, 5, and 7. *Bd* growth was estimated by calculating the slopes of optical density measurements for all wells individually, using the heat-killed negative controls as a spectrophotometer blank. Proportion of growth was measured as the ratio of slopes in experimental wells to the average slope of the nutrient-depleted positive controls. Proportion of inhibition for each experimental well was calculated by subtracting its proportion of growth from 1. To determine average proportion of inhibition for each bacterial isolate, technical replicates were averaged. Average proportions of inhibition above 80% were considered strongly inhibitory.

### Glucocorticoid quantification

We quantified levels of two glucocorticoid hormones, cortisol and corticosterone, from foam swabs (PurSwab®) stored in 100% ethanol. To begin glucocorticoid extraction, we transferred 1 mL ethanol samples and swabs (PurSwab®) from freezer vials to 18 × 150 mm borosilicate culture tubes. Freezer vials were rinsed four times with 750 µL of 100% ethanol and the contents of each wash were decanted into the same culture tubes that contained the respective swab. A volume of 2 mL of ethanol was added to bring the total volume of each culture tube to 6 mL. Samples were vortexed for 5 min to help free any remaining glucocorticoids from the swab. After vortexing, we removed swabs from culture tubes with long forceps that had been washed with ethanol, and 20 mL of distilled water was added to each tube to bring the total volume to 26 mL.

Samples were then passed slowly, under vacuum, through Hypersep C18 columns (3 cc, 500 mg bed weight, Thermo Fisher) fitted to a 24-port manifold using Tygon 2275 formulation tubing, which does not adsorb hormones or leach contaminants. We primed columns before use with 2 consecutive washes of 2 mL of methanol followed by 2 consecutive washes with 2 mL of deionized water. After passing samples through the columns, and before elution, we rinsed columns with 2 mL of deionized water to remove residual salts. We then eluted glucocorticoids from the columns with 3 consecutive 2 mL washes of ethyl acetate, which pulls the “free” (not conjugated to sulfates or glucuronides) hormone fraction from the columns^110^. A gentle stream of ultrapure nitrogen gas (~7 bar) was used to evaporate the solvent at 37 °C in a water bath under a fume hood. The remaining residue was re-suspended in 10 µL of ethanol, vortexed for 1 min, mixed with 150 µL enzyme-linked immunosorbent assay (ELISA) buffer (Cayman Chemical), and vortexed once more for 1 min. Cayman Chemical ELISA kits were used to quantify concentrations of cortisol (Catalog No. 500360, strip wells; assay sensitivity = 35 pg/mL) and corticosterone (Catalog No. 501320, strip wells; assay sensitivity = 30 pg/mL), according to the manufacturer’s procedures. Based on serial dilutions conducted to validate the assay, we determined that 1:4 was the best dilution factor for measuring both cortisol and corticosterone. Therefore, 40 µL of ethanol and 600 µL of ELISA buffer were added to bring the total resuspension volume in each tube to 800 µL (50 µL of ethanol and 750 µL of ELISA buffer). All experimental samples were run in duplicate and were arranged randomly on four ELISA plates. Cortisol and corticosterone ELISAs had minimal cross-reactivity with other natural and synthetic glucocorticoids (e.g., Corticosterone: 11-deoxycorticosterone – 15.8%, prednisolone – 3.4%, 11-dehydrocorticostone – 2.9%, cortisol – 2.5%; Cortisol: 11-deoxycorticosterone – 0.23%, prednisolone – 4.0%, cortexolone – 1.6%).

To validate the glucocorticoid extraction and quantification process, 10 µL of the resuspended hormone extract from thirty randomly selected experimental samples were combined. The combination of these samples created a pool of experimentally derived hormone that represented average *Boana faber* glucocorticoid levels. The pool was serially diluted from 1:1 to 1:128 (4 dilutions) and assayed in duplicate. The serial dilution curve was parallel to the standard curve, indicating no matrix effects (i.e., when components of the sample itself alter the chemistry of the ELISA kit and thus the validity of the assay). The pooled samples used to conduct the validation also were run in duplicate at the beginning and end of each of the four ELISA plates; the intra-assay coefficients of variation were 5.8%, 7.1%, 1.2%, and 1.5% for cortisol and 4.1%, 6.5%, 4.2%, and 6.3% for corticosterone. The inter-assay coefficients of variation were 11.7% for cortisol and 4.9% for corticosterone. For analyses, we log10 transformed cortisol and corticosterone concentrations (pg) to correct skewed distributions.

### Molecular methods

We performed DNA extractions from skin swabs (MedicalWire®) using Qiagen DNeasy kits, following the manufacturer’s protocol at the Universidade do Vale do Rio dos Sinos, São Leopoldo, RS, Brazil. We included negative controls (extraction reagents with no sample) to monitor possible contamination during extraction. Final elution volume was 100 µL, achieved through two rounds of 50 µL elution.

To identify bacterial isolates from challenge assays, single colonies from pure cultures were picked and placed in 8-strip tubes with 50 µL sterile MilliQ. After vortexing, 5 µL of bacterial suspension was added to 96-well plates containing 100 µL Chelex 100 solution [5 g Chelex 100/50 mL MilliQ] for DNA extraction. Extracted DNA was amplified using 16 S rRNA primers 907 R and 8 F^111^. Sanger sequencing of reverse strands was performed by MCLab (San Francisco, CA). Resulting sequences were trimmed to remove low-quality ends and primers using Geneious Prime.

To detect *Bd* infection and quantify *Bd* loads, we ran qPCR analysis using DNA extracted from skin swabs and diluted 1:10. We used TaqMan qPCR assays with primers targeting the ITS and 5.8 S regions^107^. To standardize quantification across runs we used gBlock synthetic *Bd* standards (IDT) ranging from 10^6^ to 10^2^ gene copies. We ran each sample in duplicate, then ran any mismatched samples in triplicate. Only samples that were positive in two of the three runs were recorded as positive. We averaged *Bd* loads for positive sample then log10 transformed load values to account for non-normal distributions characteristic of pathogen load data.

Following the Earth Microbiome Project 16 S Illumina Amplicon Protocol^112,113^, we conducted metabarcoding of the skin microbial communities, targeting the V4 region of the 16 S rRNA gene. We ran samples in duplicated using dual-indexed PCR-amplification on unique pairs of barcoded primers (515 F and 806 R) with the following recipe per duplicate pair: 12.2 µL of UltraPure water, 4 µL of 5X Phire reaction buffer (Thermo Scientific), 0.4 µL of 2.5 mM dNTPs (Invitrogen), 0.4 µL of Phire Hot Start II DNA Polymerase (Thermo Scientific), 0.5 µL each of 10 µM barcoded forward and reverse primers (IDT), and 2 µL of sample DNA. We ran 96-well PCR plates on SimpliAmp thermal cyclers (Thermo Scientific) using the following protocol: 98 °C for 3 min, 38 cycles of 98 °C for 5 s, 50 °C for 5 s, and 72 °C for 15 s, then 72 °C for 3 min before holding at 12 °C. Each plate contained a negative control in the last well consisting of water without template DNA to monitor any potential contamination of PCR reagents during preparation. After each run, we visualized PCR product on a 1% agarose gel to confirm DNA amplification then combined duplicate plates and pooled 2 µL of each amplified sample into a single amplicon library. Using the QIAquick Gel Extraction Kit (Qiagen), following manufacturer’s instructions for salt-sensitive reactions, we purified the library to remove extra primers and non-target amplicons. We sent the 16 S library for sequencing at Tufts University Core Facility (TUCF Genomics), Boston, MA, USA on an Illumina MiSeq V2 with 2 ×250 bp paired-end reads. All bacterial sequences are deposited in the NCBI Sequence Read Archive (BioProject PRJNA940133).

After receiving demultiplexed fastq files from the sequencing facility, we imported forward reads for each sample into Quantitative Insights into Microbial Ecology II (QIIME2 version 2019.10). We followed the Deblur pipeline to trim sequences to 150 bp, filter out sequences with low quality scores^114^, and cluster sequences into sub-operational taxonomic units (sOTUs) based on 97% similarity. We assigned taxonomy to sOTUs with the Greengenes 13.8 reference sequence database. This allowed us to remove chloroplast and mitochondrial sequences, and any reads from sOTUs contributing less than 0.005% of total reads^114^. We rarefied the sOTU table to 1,500 reads based on rarefaction curves (Supplementary Fig. 6), resulting in 14 of 135 samples being excluded including all extraction and PCR controls. For analyses of alpha diversity of skin bacterial communities, we calculated the sOTU richness and Shannon’s diversity for each sample. For beta diversity, we calculated Bray-Curtis dissimilarity between samples then used non-metric multidimensional scaling (nMDS) axis 1 (MDS1) for analysis.

After Sanger sequencing of bacterial isolates from challenge assays, we imported trimmed reads into QIIME2 and clustered into sOTUs at 99% sequence similarity. From the 76 isolates with *Bd* inhibitory properties, 9 unique sOTUs were clustered with sequences from our skin microbiome dataset (Supplementary Table 3). We then used sOTU identity to calculate, for each sample, the proportion of bacterial taxa in the microbiome matching isolates with putative *Bd*-inhibitory function.

### Statistics and reproducibility

To test for differences in alpha diversity, cortisol, corticosterone, and body condition over time between treatments we ran Linear Mixed Models (LMM), with individual frog ID as a random effect to account for pseudoreplication associated with repeated sampling. As not all frogs were outfitted with trackers on the same day, we used the number of days since initial release for each frog instead of Julian date as a metric of temporal comparison. For alpha bacterial diversity, we ran a generalized LMM with a Poisson distribution, adding sOTU richness as the response variable, and days since release, treatment, and their interaction as explanatory variables, then used analysis of covariance (ANCOVA) to measure differences (Diff) in slopes over time between the three treatments. For glucocorticoid levels, we ran an LMM with normal residual distribution, adding log10-transformed cortisol or corticosterone as the response variable, and days since release, treatment, and their interaction as predictors, and used ANCOVA to measure differences in slopes between the three treatments. To test for the effect of treatment on host body condition (Mass/SVL), we ran an LMM with normal residual distribution, controlling for individual frog ID and days since release by including frog ID as a random effect, then nesting days since release within frog ID, and we followed this with an HSD *post hoc* test to compare averages.

To test for an effect of transmitter attachment on microbiome diversity and functional potential, we compared Continuous-Control (transmitter) frogs to resident (non-transmitter) frogs sampled at the same site. We only used Continuous-Control frogs to avoid confounding stressors of translocation. This resulted in 23 transmitter samples and 17 non-transmitter samples. We ran analysis of variance (ANOVA) to test for differences in cortisol, corticosterone, and body condition between transmitter and non-transmitter frogs over time. We use Julian date for each frog as a metric of temporal comparison to accurately match transmitter frogs with their non-transmitter equivalents. For each model, we added either sOTU richness or proportion of *Bd*-inhibitory bacteria as the response and transmitter attachement, Julian date, and their interaction as predictors.

We used the linear discriminant analysis effect size method on the galaxy platform (https://huttenhower.sph. harvard.edu/galaxy/) to detect significantly differentially abundant bacterial sOTUs between frogs pre- and post-translocation among the three treatment groups: Continuous-Control, Continuous-Translocated, and Fragment-Translocated. We separately included all frog captures post-translocation for this analysis. We increased the threshold LDA score to 3, but otherwise maintained default parameters. Using the heatmap.2 function from the gplots package in R^115,116^, we created a plot to visualize differentially abundant sOTUs based on the linear discriminant analysis effect size results.

To measure changes in microbiome community composition and host movement over time we used community trajectory analysis (CTA). This analysis uses multidimensional space to map the trajectory, or direction of multidimensional movement, of microbial community composition between sequential sampling points. As trajectory analyses require at least three sequential points, we reduced our dataset to include only frogs with 3 or 4 sample points and removed any points after the fourth sampling timepoint as there were too few samples across all treatments. After this filtering, we were left with 5 frogs in the Continuous-Control treatment, 4 frogs in the Continuous-Translocated treatment, and 9 frogs in the Fragment-Translocated treatment. We used nMDS to summarize microbiome community data for CTA, using Bray-Curtis distances (k = 3, stress = 0.15). We used the ecotraj package in R^80,117^ to calculate lengths (trajectoryLength function), angles (trajectoryAngles function), and overall directionality microbiome composition shifts (trajectoryDirectionality function) for each frog (Supplementary data 3). Segment length is measured for each pair of sequential points, with higher values indicating greater change in the microbial community between those points. Trajectory angle (0°–180°) is measured for each pair of segments (e.g., sample 1–2 and sample 2–3), with lower values (closer to 0°) indicating little shift in direction of the microbial community composition while higher values (closer to 180°) indicate a greater change in direction often associated with a disturbance event occurring between two segments. Overall directionality accounts for the changes in angles across the entire trajectory and is higher (closer to 1) when the microbial community shift is consistently following the same direction in multidimensional space, which indicates that the community is undergoing a change in composition state^80,81^. We used one-way analysis of variance (ANOVA) to test for significant differences in mean angles and directionality between treatments. We also used ANOVA to test for significant differences in segment length for each treatment fit separately. We used HSD test to measure differences between segments. To compare whole trajectories between treatments, we averaged the microbiome community data by treatment and time point resulting in 4 equivalent time points for each treatment. We again used nMDS to summarize microbiome community data for trajectory analysis (k = 2, stress = 0.07).

To analyze how habitat resistance, which is a quantitative estimate of how environmental parameters affect animal movement^118^, influences microbiome composition, we first mapped land cover based on nine classes (forest, waterway, open habitat, pastureland, isolated trees, silviculture, wetland, roads, and human construction) using a supervised classification from SPOT6 images at 1.5 meter resolution. We combined our maps with the drainage mapping available at the Brazilian Sustainable Development Foundation (available at http://geo.fbds.org.br/). We also created a 10-meter buffer around forested areas to differentiate forest edge from interior forest. Land cover mapping was then reclassified to 6 six classes (forest, forest edge, non-forested areas, wetland within forest edge, wetland in core forest areas, and wetland in open areas; Supplementary Fig. 7). Wetland classes combine waterways, drainages, and swamp. Non-forested areas combine all anthropogenic and non-forested habitats (open habitat, pasture, human construction, roads, etc.). From our land use map, we constructed a resistance matrix, where low resistance values denote permeability to movement and high resistance values denote barriers to movement^118^. Forested areas (1), wetlands on forest edges (5), wetlands in open areas (10), dry forest edges (10), and forest edges (10) presented low and medium resistance, while non-forested areas (100) present high resistance to movement. Using Circuitscape 4.0^119^ in pairwise modeling mode, we estimate the average resistance between each pair of movement points, for each individual. Analyses were done using Fragstats version 4.2.1^120^ and ArcGIS version 10.8.1^121^. We used the enmSdm packagee (pointDist function)^122^ in R to calculate a distance matrix between capture GPS points (±5 m error). We used the same subset of samples used in CTA, but also excluded initial swabs for translocated treatments as we did not have resistance measurements across the entire landscape, and this distance would dramatically inflate resistance values. We then ran GLMs to compare segment lengths of microbiome trajectories (from CTA) to both average resistance values and host movement distances for each treatment in turn. For each model, we added microbiome trajectory segment length as the response and average resistance and movement distance as predictors.

We used principal component analysis (PCA) and factor analysis to ensure selection of explanatory variables that were not highly correlated for later models (Fig. 6; Supplementary Table 4). Additionally, factor analysis reveals underlying relationships between variables that can assist with interpretation of patterns. Due to the enzootic prevalence (15%) and relatively low infection loads (108 ± 464 gene copies) of *Bd* in this system, and significant correlation between *Bd* infection loads and proportion of *Bd*-inhibitory bacterial taxa in the microbiome (Fig. 6), we excluded data on *Bd* from our multivariate mixed models. Similarly, we excluded MDS1 from our analysis as it loaded with sOTU richness (Fig. 6). Cortisol and corticosterone also loaded together, so we analyzed these two glucocorticoids separately in our models to parse out the differences associated with each hormone without creating multicollinearity.

To analyze the direct and indirect relationships between variables we used piecewise Structural Equation Models (psem argument, piecewiseSEM package in R)^123^ with individual linear mixed effects models (lme argument, nlme package in R)^124^. We ran models separately for each treatment, including body condition (mass/SVL), cortisol, corticosterone, sOTU richness, and inhibitory proportion in each model. All variables were centered and standardized prior to analysis. We included individual frog as a random effect to control for repeated measures. We also controlled for temporal autocorrelation by including the number of days since initial release in a correlation structure argument (corAR1 argument, nlme package in R). Further details on our analytical pipeline can be found as supplemental information (Supplementary software 1; Supplementary data 4).

We measured within-individual (between sequential sampling points) variation, also referred to as repeatability, for sOTU richness and proportion of *Bd*-inhibitory bacteria. We conducted this analysis using the asreml package in R (https://www.vsni.co.uk/software/asreml-r/), subsetting the dataset to include only individuals with at least one recapture. All variables for the resulting 26 individuals (Continuous-Control = 5, Continuous-Translocated = 7, and Fragment-Translocated = 14) were centered and standardized to have a mean of zero and standard deviation of one prior to analysis. This analysis enables us to identify whether fluctuations in each variable are mainly driven by factors that are intrinsic or extrinsic to the host, providing insight into the consistency of differences between individuals^88^.

All statistical analyses were run in R v. 4.2.2^115^ and JMP v. 15^125^, unless otherwise specified. The full dataset used for analyses is available online (Supplementary data 5 and 6).

### Reporting summary

Further information on research design is available in the Nature Portfolio Reporting Summary linked to this article.

### Supplementary information

Supplementary Information

Supplementary Software 1

Supplementary Data 1

Supplementary Data 2

Supplementary Data 3

Supplementary Data 4

Supplementary Data 5

Supplementary Data 6

Reporting Summary

## Data Availability

We have included raw data used for statistical analyses and construction of Figs. 2 and 3 as a supplemental file (Supplementary data 3 and Supplementary Data 5). We have also included data generated from trajectory analyses and the habitat resistance analysis which was used to generate Figs. 4 and 5 (Supplementary data 1). All bacterial sequences are deposited in the NCBI Sequence Read Archive (BioProject PRJNA940133). Any additional data are available upon request.

## Code availability

Code generated in in R v. 4.2.2. to run the path analyses has been included as a supplemental file (Supplementary software 1), along with the associated data file (Supplementary data 2). Any additional code is available upon request.
