# Supplementary material for: Linking microbiome and stress hormone responses in wild tropical treefrogs across continuous and fragmented forests
Source: Commun Biol. 2023 Dec 12;6:1261. doi: 10.1038/s42003-023-05600-9 (PMC10716138; doi:10.1038/s42003-023-05600-9)

Supplementary Materials

Linking microbiome and stress hormone responses in wild tropical treefrogs across continuous and fragmented forests

Wesley J. Neely, Renato A. Martins, Camila M. Mendonça da Silva, Tainá Ferreira da Silva, Lucas E. Fleck, Ross D. Whetstone, Douglas C. Woodhams, W. Harrison Cook, Paula R. Prist, Victor H. Valiati, Sasha E. Greenspan, Alexandro M. Tozetti, Ryan L. Earley, and C. Guilherme Becker

Supplementary note 1

Bromeliads were a common site of capture in this study, with 20% of frogs recaptured in bromeliads (Supplementary table 1; Supplementary figure 1). This was a somewhat unexpected finding, as few studies have recorded this species using bromeliad tanks as shelter [1]. These epiphytes contain small reservoirs of water that could allow pathogen amplification through exponential reinfection with free-swimming zoospores [2]. During times of low rainfall, frogs may be more likely to seek out these humid refugia and subsequently intensify population-wide infection prevalence during large breeding events [3,4]. A consequence of frog clustering during breeding events is increased predation, although individual predation risk is reduced in large choruses [5]. *Boana faber* is a relatively short-lived species and thus likely places more investment in reproductive success over predator avoidance during breeding [6]. For this reason, we expected to record natural predation during our study, and found evidence of nine such events. Most of the frogs predated upon at continuous forest sites were likely eaten by a crab-eating fox (*Cerdocyon thous*). Multiple individuals of this species were seen using the trails where four trackers from site #3 were recovered and they are one of few predators in the area that could carry frogs 900 m before eating them. In addition to the 9 radio-tagged frogs that were predated upon, 15 additional trackers went missing. These missing individuals may have been predated upon and had their transmitters broken, predated upon by wading birds and moved out of range, or may have moved out of range on their own.

Supplementary References

1. Greenspan, S. E. *et al.* Arthropod–bacteria interactions influence assembly of aquatic host microbiome and pathogen defense. *Proceedings of the Royal Society B: Biological Sciences* **286**, 20190924 (2019).
2. Lacerda, J. V. A. *et al.* Anurans in bromeliads, Parque Estadual da Serra do Brigadeiro, State of Minas Gerais, southeastern Brazil. *Check List* **5**, 800. (2009).
3. Ruggeri, J. *et al.* Seasonal prevalence of the amphibian chytrid in a tropical pond-dwelling tadpole species. *Diseases of Aquatic Organisms* **142**, 171–176 (2020).
4. Ruggeri, J., De Carvalho-E-silva, S. P., James, T. Y. & Toledo, L. F. Amphibian chytrid infection is influenced by rainfall seasonality and water availability. *Diseases of Aquatic Organisms* **127**, (2018).
5. Ryan, M. J., Tuttle, M. D. & Taft, L. K. The costs and benefits of frog chorusing behavior. *Behav Ecol Sociobiol* **8**, 273–278 (1981).
6. Haddad, C. F. B. *et al.* *Guide to the Altantic forest amphibians: diversity and biology*. (Anolis Books, 2013).

Supplementary tables

Supplementary table 1: Percentages of different capture locations for recaptured frogs. Habitat is denoted by the following letters: W = in water body, G = on ground, T = on tree branch, and B = in bromeliad. All frogs were initially captured in the water. Bolded values show most common habitats for each treatment.

Treatment	W	G	T	B
Continuous-Control	5.6%	**9.7%**	**11.1%**	4.2%
Continuous-Translocated	1.4%	**13.9%**	4.2%	5.6%
Fragment-Translocated	4.2%	9.7%	12.5%	**18.1%**
All Continuous	6.9%	**23.6%**	15.3%	9.7%
All	11.1%	**33.3%**	**27.8%**	**27.8%**

Supplementary table 2: Averages and standard deviations for lengths of microbial community trajectory paths (L), trajectory directionality (Dir), and mean trajectory angles (A) for each treatment. Number of frogs in each treatment is given (N).

Treatment	N	L	Dir	A
Continuous-Control	5	0.600 ± 0.307	0.433 ± 0.042	98.064 ± 9.333
Continuous-Translocated	4	0.719 ± 0.275	0.406 ± 0.047	102.499 ± 8.766
Fragment-Translocated	9	0.664 ± 0.270	0.405 ± 0.057	105.348 ± 11.543

Supplementary table 3: Taxonomy of *Bd*-inhibitory isolates. Isolate reads across the rarefied microbiome dataset are also given.

**ID**	**Phylum**	**Class**	**Order**	**Family**	**Genus**	**Species**	**Reads**
1	Bacteroidetes	Flavobacteriia	Flavobacteriales	[Weeksellaceae]	*Chryseobacterium*		25566
2	Proteobacteria	Gammaproteobacteria	Pseudomonadales	Pseudomonadaceae	*Pseudomos*	*fragi*	16079
3	Proteobacteria	Gammaproteobacteria	Xanthomonadales	Xanthomonadaceae	*Stenotrophomos*		8872
4	Proteobacteria	Gammaproteobacteria	Enterobacteriales	Enterobacteriaceae			8688
5	Proteobacteria	Gammaproteobacteria	Pseudomonadales	Pseudomonadaceae	*Pseudomos*	*veronii*	3188
6	Proteobacteria	Gammaproteobacteria	Enterobacteriales	Enterobacteriaceae	*Pantoea*		1700
7	Proteobacteria	Gammaproteobacteria	Enterobacteriales	Enterobacteriaceae			1668
8	Actinobacteria	Actinobacteria	Actinomycetales	Microbacteriaceae			244
9	Actinobacteria	Actinobacteria	Actinomycetales	Microbacteriaceae			51

Supplementary table 4: Rotated factor loadings for principal components analysis on *Bd* infection loads, body condition, cortisol, CORT, sOTU richness, MDS1, and inhibitory proportion. Bolded values highlight variables that are significantly loading for each factor.

Component	Factor 1	Factor 2	Factor 3
Bd infection loads	-0.069	0.069	**0.579**
Body condition	0.162	-0.265	-0.137
Cortisol	0.154	**0.704**	**-0.485**
CORT	-0.108	**0.855**	0.153
sOTU richness	**0.950**	-0.020	-0.100
MDS1	**0.933**	-0.146	-0.045
Inhibitory proportion	-0.034	0.031	**0.807**

Supplementary figures

Supplementary figure 1: Timeline of *Bd* infection status and habitat use for frogs across treatments. Timelines for each frog are grouped by treatment. Frog ID numbers match those in Supplementary data 5. Habitat at time of capture is denoted by the following letters: W = in water body, G = on ground, T = on tree branch, and B = in bromeliad. Black filled circles indicate *Bd* positive timepoints and grey filled circles indicate *Bd* negative timepoints.

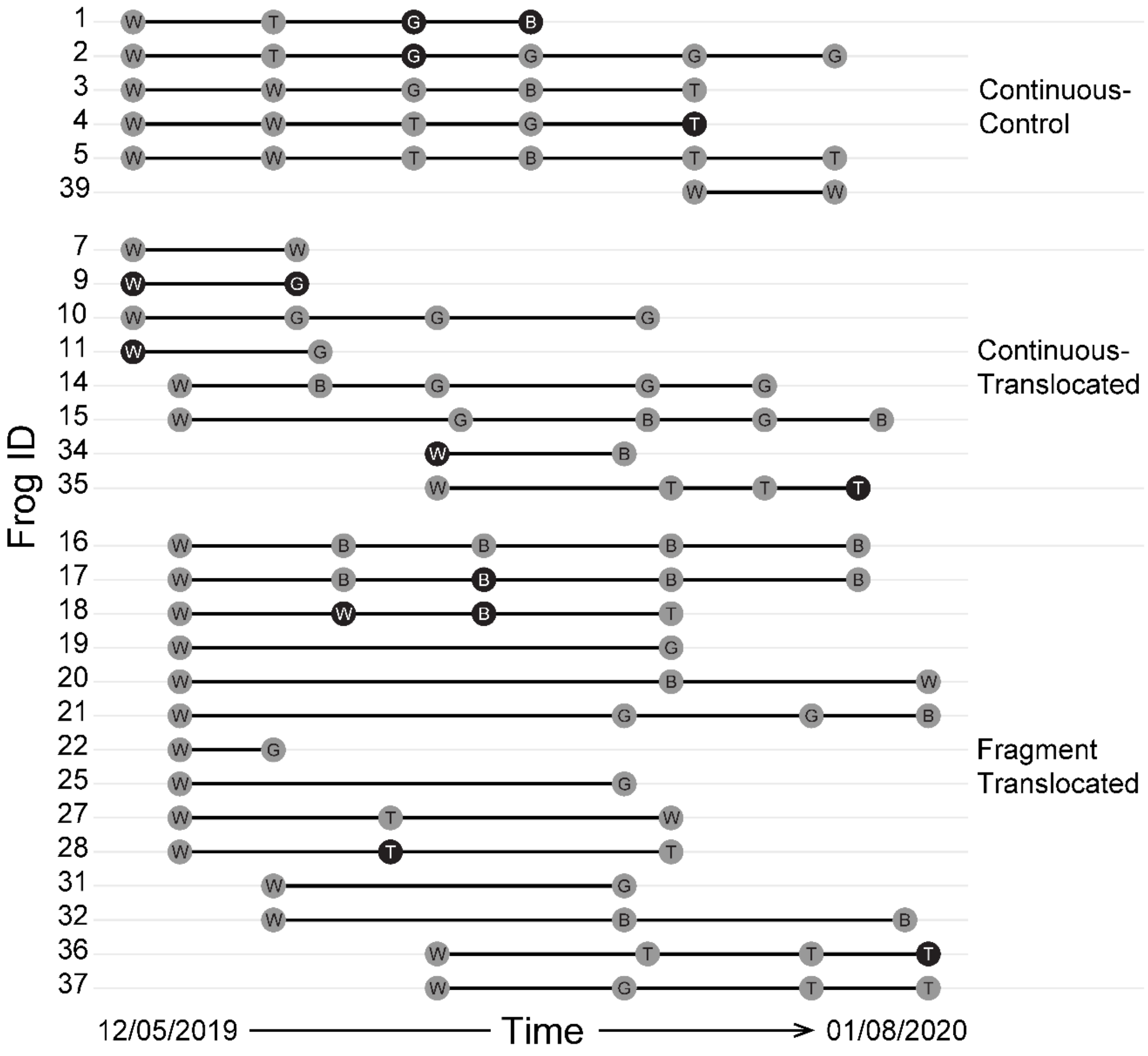

Supplementary figure 2: Changes in frequencies of bacterial taxa over time. Bar plots show the relative frequency of the dominant phyla (a) and dominant sOTUs (b) of bacteria over time. Changes in dominant phyla are separated by treatment, while changes in dominant sOTUs are shown for all treatments together.

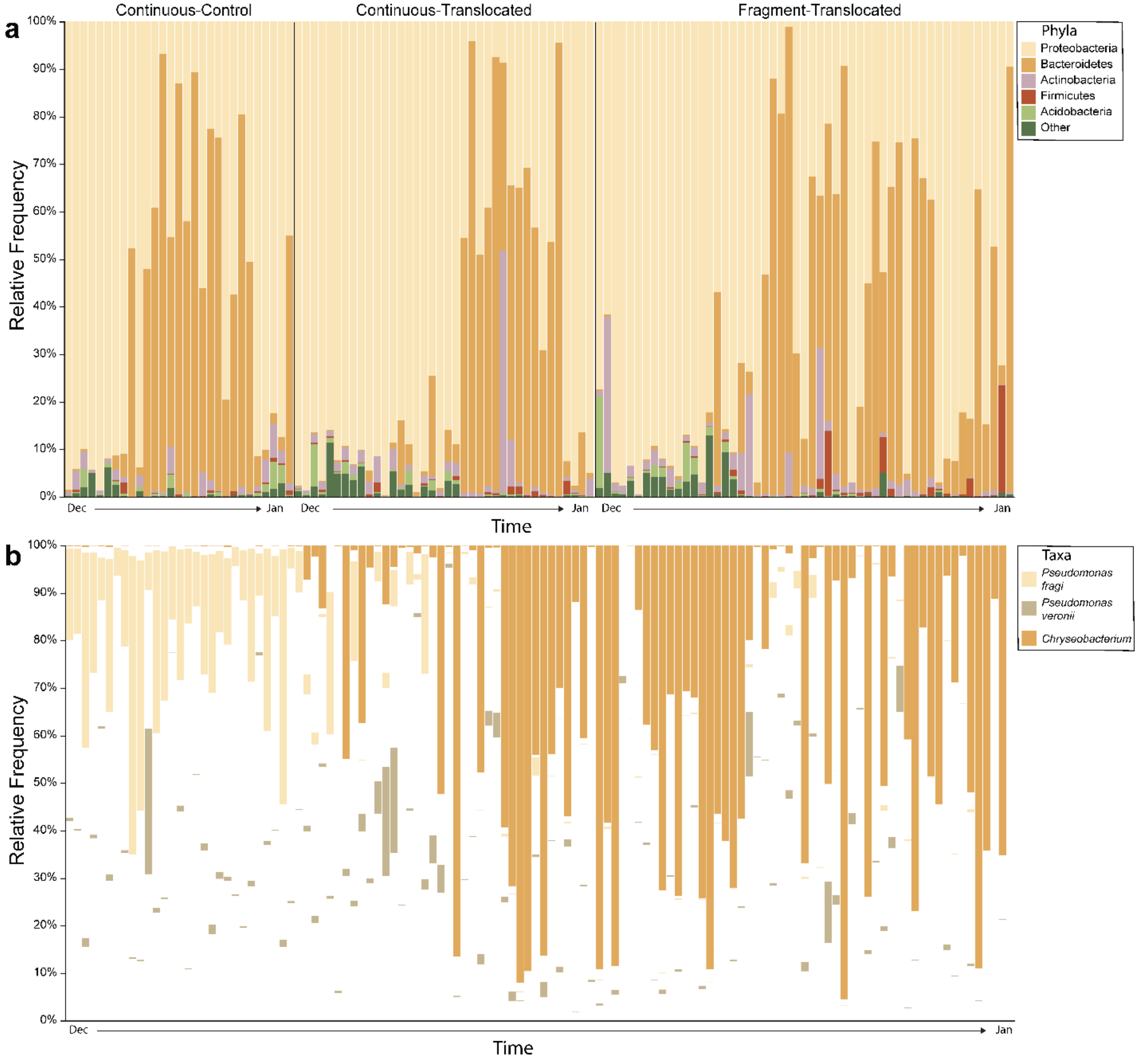

Supplementary figure 3: Relative abundance of differentially abundant sOTUs pre and post translocation among our three treatment groups. Each column represents and individual frog. Numbered rows correlate to sOTU taxonomy in Supplementary data 1. *Bd*-inhibitory isolates are shown with dashed outlines and correspond to taxonomy in Supplementary table 3: 87 = inhibitory isolate 4 (Enterobacteriaceae), 91 = inhibitory isolate 2 (*Pseudomonas fragi*), 97 = inhibitory isolate 3 (*Stenotrophomonas*). Gradient colors show relative abundance of each taxon ranging from 0 to 1.

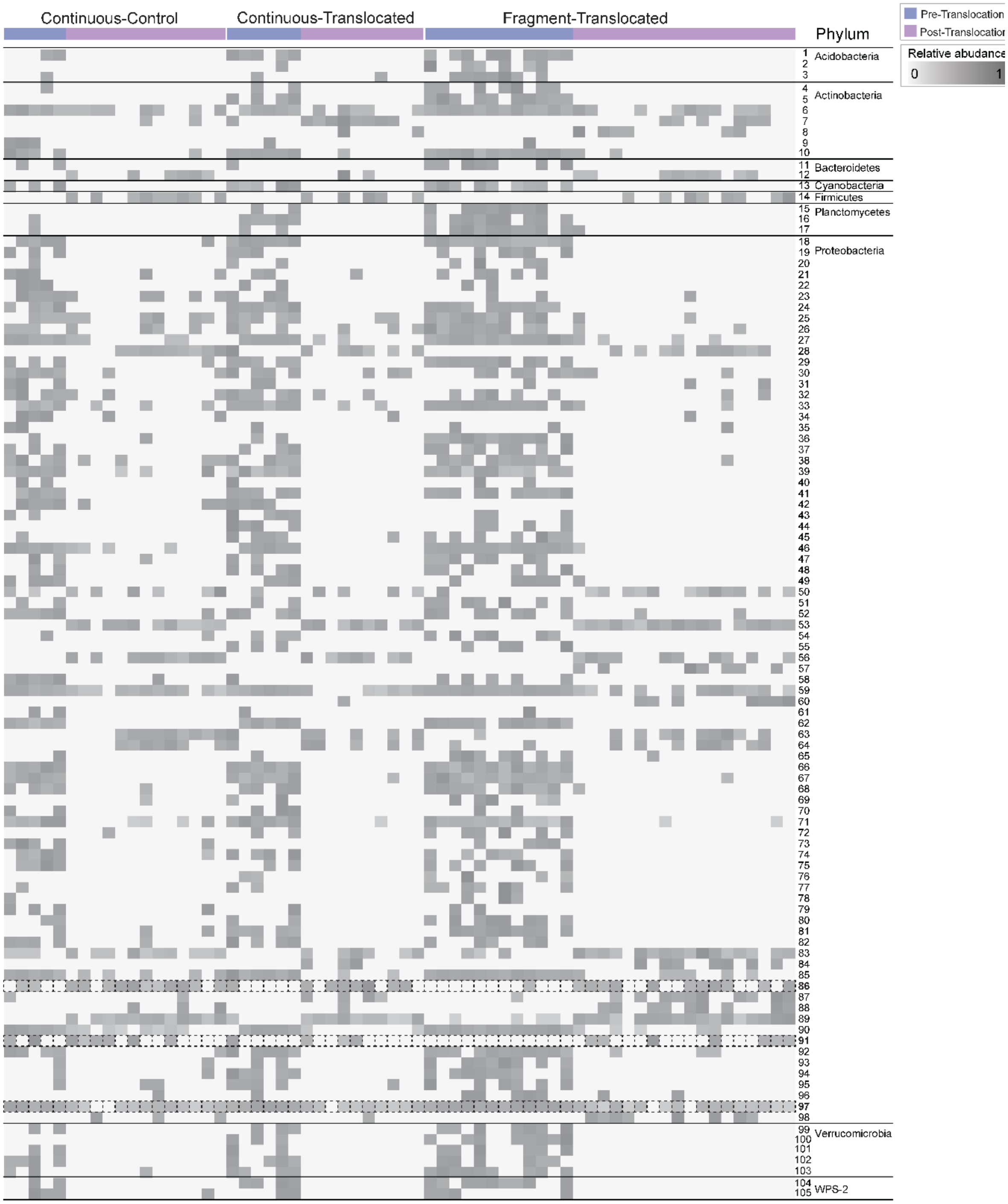

Supplementary figure 4: Shifts in microbiome community composition over time between treatments for each individual. Plots show shifts in individual microbiome composition trajectories over time, with centroids averaged for each timepoint within each treatment. The three treatments are Continuous-Control (green, n=5), Continuous-Translocated (orange, n=4), and Fragment-Translocated (purple, n=9). Ordination is based on non-metric multidimensional scaling with Bray-Curtis distances.

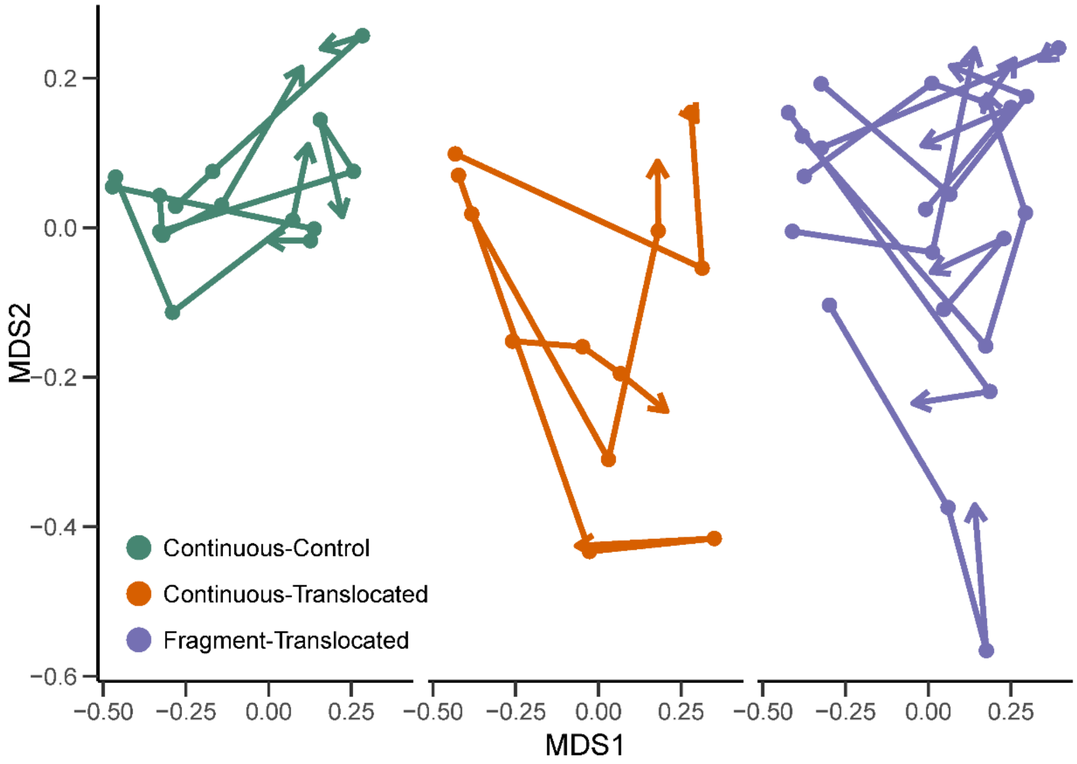

Supplementary figure 5: Seasonal weather dynamics from historical records and our study year. Monthly averages are shown for historical (grey) and study year (black) data for rainfall (a), temperature (b), and humidity (c). As our study was conducted in December and January, Only averages from October through March are shown. Historical data represents the three years preceding our study (2016–2019). These data were collected from the weather station in Cambará do Sul, RS, Brazil (https://tempo.inmet.gov.br/TabelaEstacoes/A001).

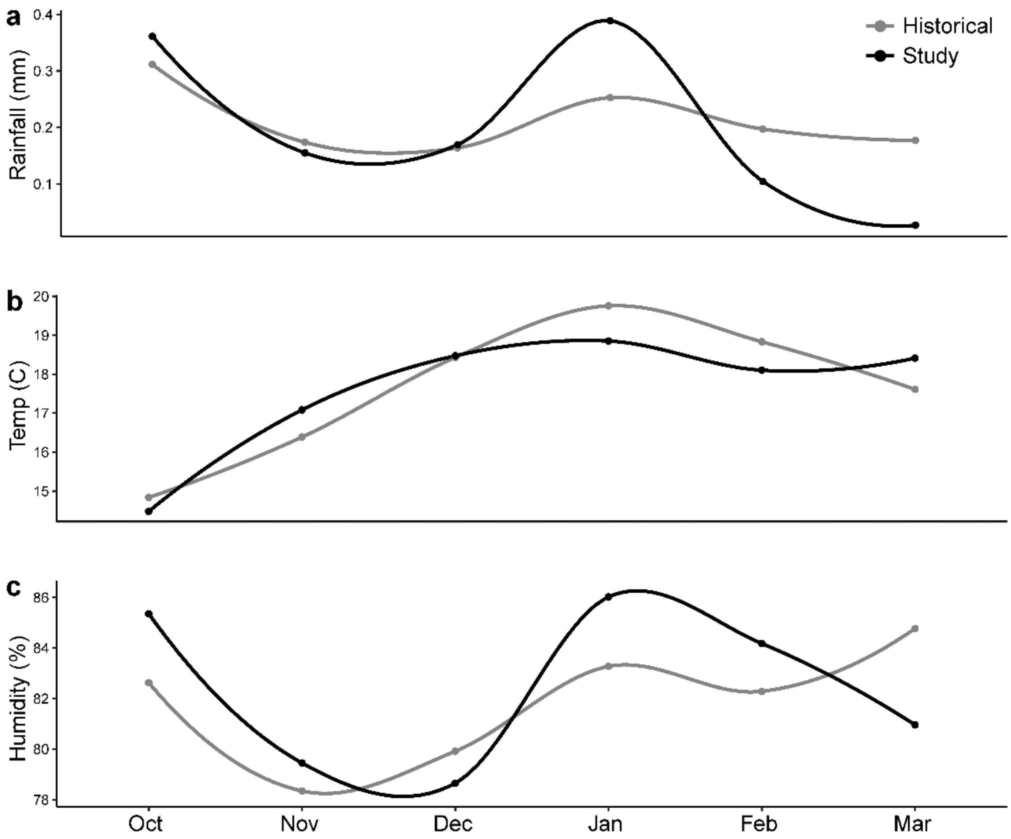

Supplementary figure 6: Rarefaction curves for microbiome data. Sequencing depth cutoff was 1,500 reads. Each line represents one sample.

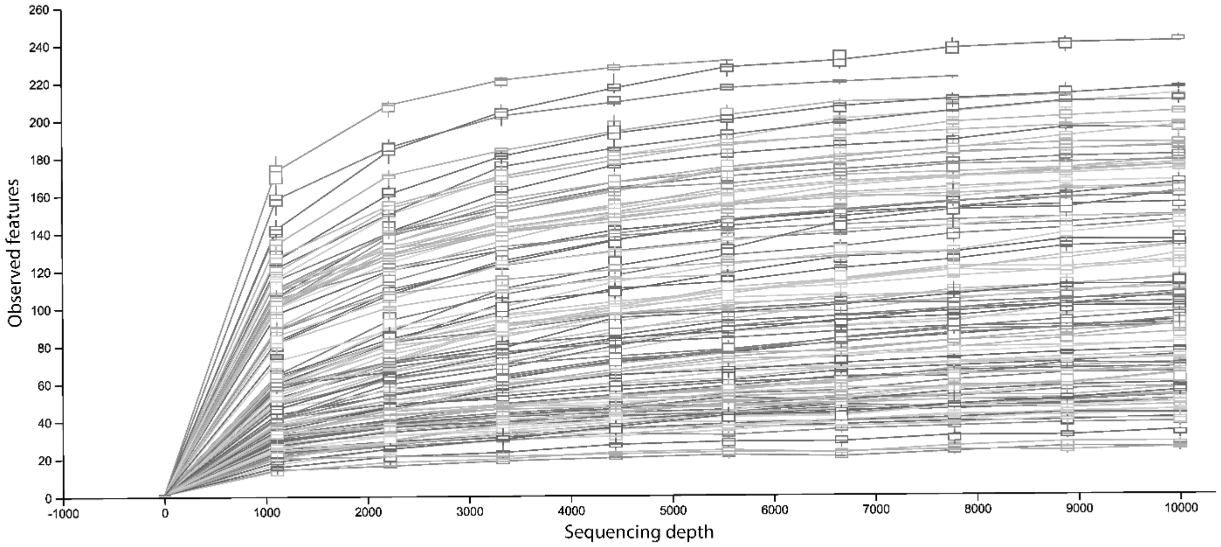

Supplementary figure 7: Maps detailing landcover at each sampling location. Land cover is classified as forest (green), forest edge (light green), non-forested areas (tan), wetland within forest edge (blue), wetland in core forest areas (dark blue), and wetland in open areas (light blue). Numbers match those in Figure 1 of the main text: Continuous-Control (f), Continuous-Translocated (d–e), and Fragment-Translocated (a–c). Each frame is 1 km wide.

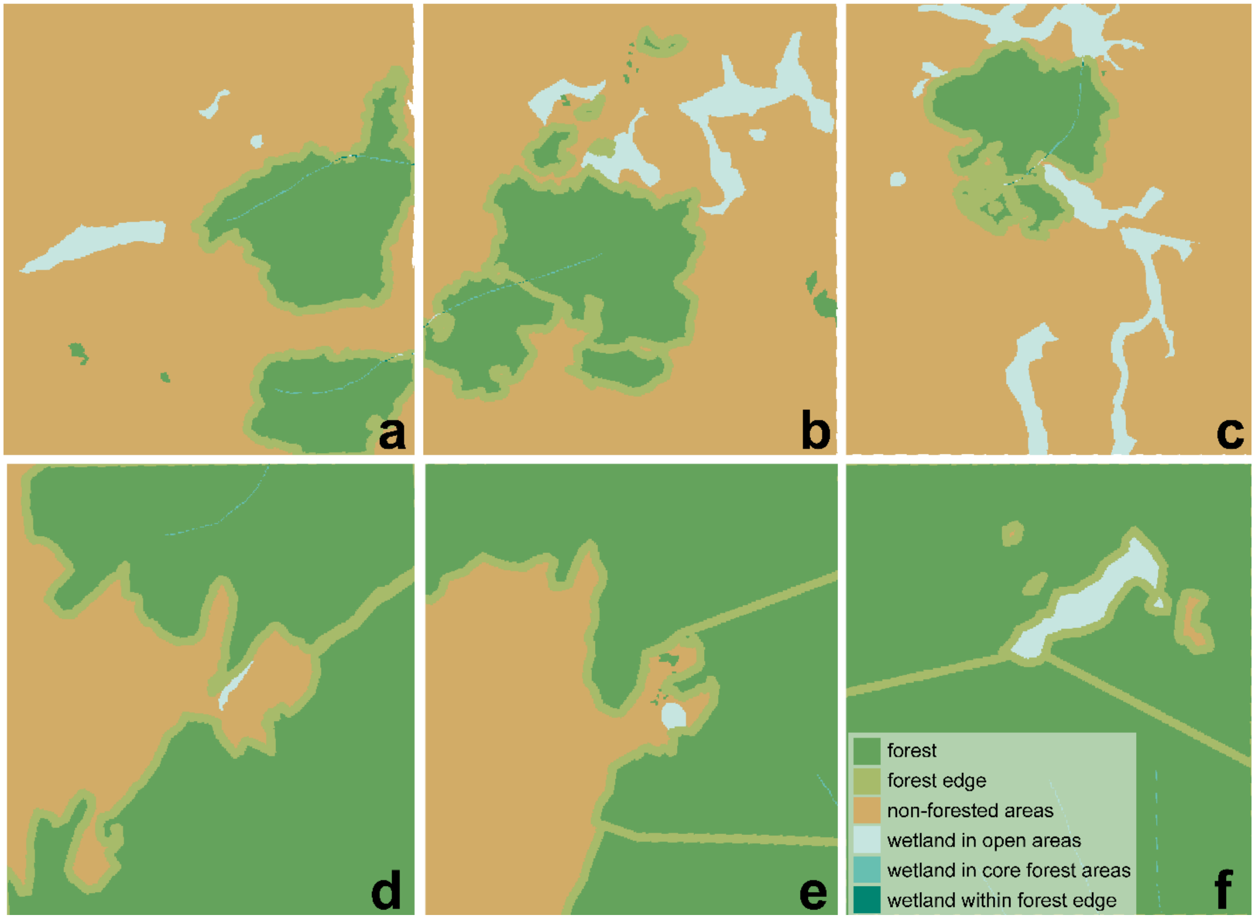

Supplement: Supplementary file 1 — Supplementary Information [file 42003_2023_5600_MOESM1_ESM.pdf]
