# Supplementary material for: Linking microbiome and stress hormone responses in wild tropical treefrogs across continuous and fragmented forests
Source: Commun Biol. 2023 Dec 12;6:1261. doi: 10.1038/s42003-023-05600-9 (PMC10716138; doi:10.1038/s42003-023-05600-9)
Supplement: Supplementary file 2 — Supplementary Software 1 [file 42003_2023_5600_MOESM2_ESM.pdf]

Path Analyses

Wesley J. Neely

2023-07-25

SetUp

Libraries

```
library(tidyverse)
```

```
## Warning: package 'tidyverse' was built under R version 4.2.3
```

```
## Warning: package 'ggplot2' was built under R version 4.2.3
```

```
## Warning: package 'tibble' was built under R version 4.2.3
```

```
## Warning: package 'tidyr' was built under R version 4.2.3
```

```
## Warning: package 'readr' was built under R version 4.2.3
```

```
## Warning: package 'purrr' was built under R version 4.2.3
```

```
## Warning: package 'dplyr' was built under R version 4.2.3
```

```
## Warning: package 'forcats' was built under R version 4.2.3
```

```
## Warning: package 'lubridate' was built under R version 4.2.3
```

```
## ── Attaching core tidyverse packages ──────────────────────── tidyverse 2.0.0 ──
## ✓ dplyr     1.1.2     ✓ readr     2.1.4
## ✓ forcats   1.0.0     ✓ stringr   1.5.0
## ✓ ggplot2   3.4.2     ✓ tibble    3.2.1
## ✓ lubridate 1.9.2     ✓ tidyr     1.3.0
## ✓ purrr     1.0.1
## ── Conflicts ────────────────────────────────────────── tidyverse_conflicts() ──
## ✕ dplyr::filter() masks stats::filter()
## ✕ dplyr::lag()    masks stats::lag()
## i Use the ]8;;http://conflicted.r-lib.org/conflicted package]8;; to force all conflicts to become errors
```

```
library(piecewiseSEM)
```

```
##
##   This is piecewiseSEM version 2.1.0.
##
##
##   Questions or bugs can be addressed to <LefcheckJ@si.edu>.
```

```
library(nlme)
```

```
##
## Attaching package: 'nlme'
##
## The following object is masked from 'package:dplyr':
##
##     collapse
```

Data Management

```
setwd("C://Users/Wesley/OneDrive - The University of Alabama/PhD Research/Brazil Radio-tracking project/Data for analysis/Pa
th Analyses/")
data <- read_csv("PathData.csv")
```

```
## Rows: 93 Columns: 16
## ── Column specification ────────────────────────────────────────────────────────
## Delimiter: ","
## chr  (1): Sex
## dbl (15): Frog_ID, Swab_num, Group, Days_Since_Release, Landscape_3way, Bd_S...
##
## ℹ Use `spec()` to retrieve the full column specification for this data.
## ℹ Specify the column types or set `show_col_types = FALSE` to quiet this message.
```

```
data_Cont <- filter(data, Landscape_3way == 0)
data_ContTrans <- filter(data, Landscape_3way == 1)
data_Frag <- filter(data, Landscape_3way == 2)
```

Path Models

Continuous-Control

```
path.mod1 <- psem(
  mod1 <- lme(Inhib_STD ~ Cortisol_STD + CORT_STD + Body_condition_STD, random=~1|Frog_ID, correlation=corAR1(0, form = ~Day
s_Since_Release|Frog_ID), data=data_Cont),
  mod2 <- lme(otus_STD ~ Cortisol_STD + CORT_STD + Body_condition_STD, random=~1|Frog_ID, correlation=corAR1(0, form = ~Days
_Since_Release|Frog_ID), data=data_Cont),
  mod3 <- lme(Cortisol_STD ~ Body_condition_STD, random=~1|Frog_ID, correlation=corAR1(0, form = ~Days_Since_Release|Frog_I
D), data=data_Cont),
    mod4 <- lme(CORT_STD ~ Body_condition_STD, random=~1|Frog_ID, correlation=corAR1(0, form = ~Days_Since_Release|Frog_ID),
data=data_Cont),
  Inhib_STD %~~% otus_STD,
  Cortisol_STD %~~% CORT_STD
)
summary(path.mod1)
```

```
##
## Structural Equation Model of path.mod1
##
## Call:
##   Inhib_STD ~ Cortisol_STD + CORT_STD + Body_condition_STD
##   otus_STD ~ Cortisol_STD + CORT_STD + Body_condition_STD
##   Cortisol_STD ~ Body_condition_STD
##   CORT_STD ~ Body_condition_STD
##   Inhib_STD ~~ otus_STD
##   Cortisol_STD ~~ CORT_STD
##
##     AIC      BIC
##  48.000   78.194
##
## ---
## Tests of directed separation:
##
##  No independence claims present. Tests of directed separation not possible.
##
## Global goodness-of-fit:
##
##   Fisher's C = 0 with P-value = 1 and on 0 degrees of freedom
##
## ---
## Coefficients:
##
##         Response          Predictor Estimate Std.Error DF Crit.Value P.Value
##        Inhib_STD       Cortisol_STD  -0.1697    0.2078 18    -0.8168  0.4247
##        Inhib_STD           CORT_STD   0.2492    0.1838 18     1.3559  0.1919
##        Inhib_STD Body_condition_STD   0.0754    0.2074 18     0.3635  0.7204
##         otus_STD       Cortisol_STD   0.4174    0.2445 18     1.7068  0.1050
##         otus_STD           CORT_STD  -0.0087    0.2419 18    -0.0359  0.9718
##         otus_STD Body_condition_STD  -0.0183    0.1992 18    -0.0921  0.9277
##     Cortisol_STD Body_condition_STD   0.1362    0.2272 20     0.5998  0.5554
##         CORT_STD Body_condition_STD  -0.0374     0.204 20    -0.1833  0.8564
##      ~~Inhib_STD         ~~otus_STD  -0.1703         - 26    -0.8286  0.2079
##   ~~Cortisol_STD         ~~CORT_STD   0.6371         - 26     3.9644  0.0003
##   Std.Estimate
##        -0.1697
##         0.2492
##         0.0754
```

```
##         0.4174
##        -0.0087
##        -0.0183
##         0.1362
##        -0.0374
##        -0.1703
##         0.6371 ***
##
##   Signif. codes:  0 '***' 0.001 '**' 0.01 '*' 0.05
##
## ---
## Individual R-squared:
##
##       Response method Marginal Conditional
##      Inhib_STD   none     0.04        0.57
##       otus_STD   none     0.15        0.16
##   Cortisol_STD   none     0.02        0.18
##       CORT_STD   none     0.00        0.00
```

```
plot(path.mod1)
```

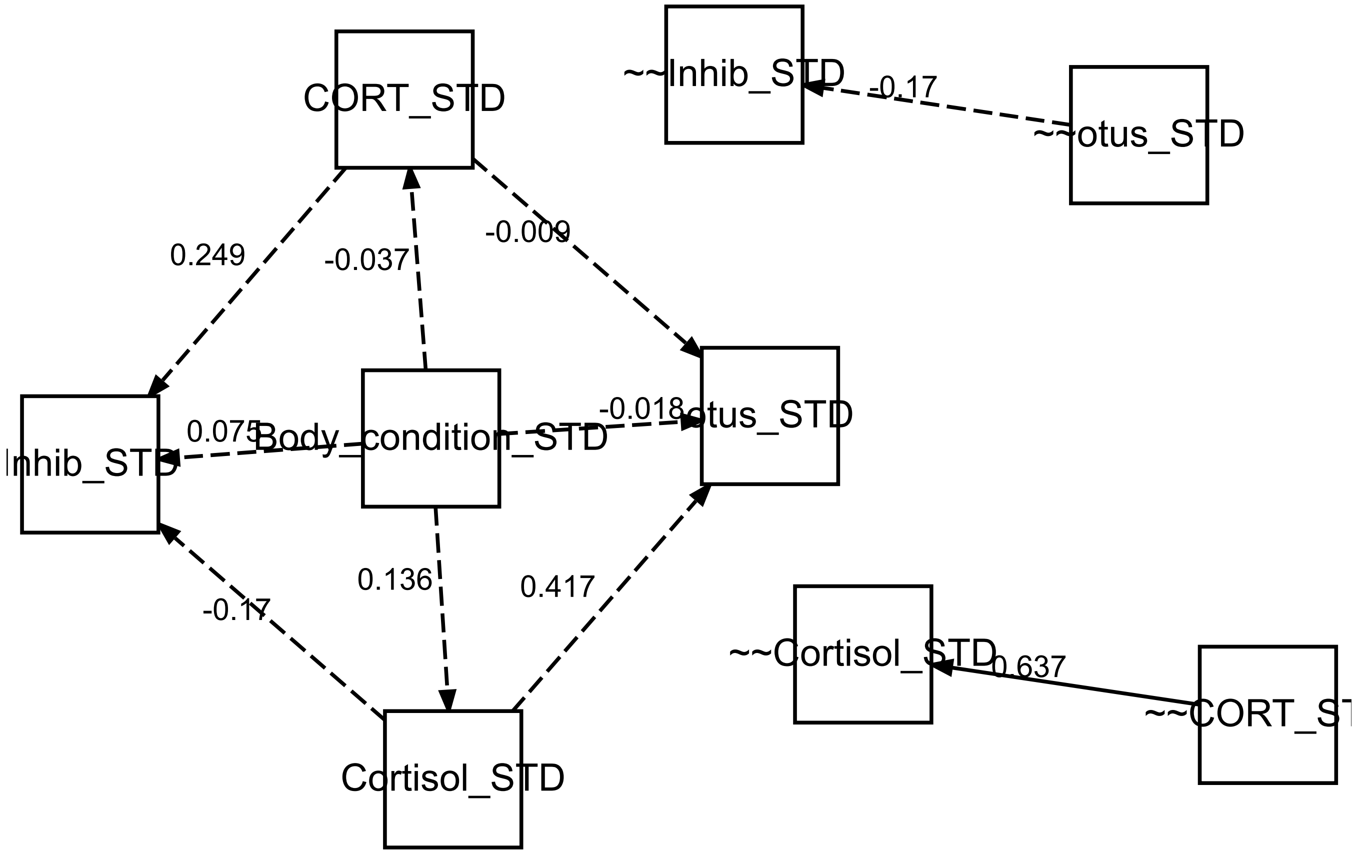
CORT_STD
~~Inhib_STD
-0.17
~~otus_STD
0.249
-0.037
-0.009
Inhib_STD
0.075
Body_condition_STD
-0.018
otus_STD
0.136
0.417
-0.17
~~Cortisol_STD
0.637
~~CORT_ST
Cortisol_STD

Continuous-Translocated

```
path.mod2 <- psem(
  mod1 <- lme(Inhib_STD ~ Cortisol_STD + CORT_STD + Body_condition_STD, random=~1|Frog_ID, correlation=corAR1(0, form = ~Days_Since_Release|Frog_ID), data=data_ContTrans[-c(23,24),]),
  mod2 <- lme(otus_STD ~ Cortisol_STD + CORT_STD + Body_condition_STD, random=~1|Frog_ID, correlation=corAR1(0, form = ~Days_Since_Release|Frog_ID), data=data_ContTrans[-c(23,24),]),
  mod3 <- lme(Cortisol_STD ~ Body_condition_STD, random=~1|Frog_ID, correlation=corAR1(0, form = ~Days_Since_Release|Frog_ID), data=data_ContTrans[-c(23,24),]),
    mod4 <- lme(CORT_STD ~ Body_condition_STD, random=~1|Frog_ID, correlation=corAR1(0, form = ~Days_Since_Release|Frog_ID), data=data_ContTrans[-c(23,24),]),
  Inhib_STD %~~% otus_STD,
  Cortisol_STD %~~% CORT_STD
)
summary(path.mod2)
```

```
##
## Structural Equation Model of path.mod2
##
## Call:
##   Inhib_STD ~ Cortisol_STD + CORT_STD + Body_condition_STD
##   otus_STD ~ Cortisol_STD + CORT_STD + Body_condition_STD
##   Cortisol_STD ~ Body_condition_STD
##   CORT_STD ~ Body_condition_STD
##   Inhib_STD ~~ otus_STD
##   Cortisol_STD ~~ CORT_STD
##
##     AIC      BIC
##  48.000   74.185
##
## ---
## Tests of directed separation:
##
##  No independence claims present. Tests of directed separation not possible.
##
## Global goodness-of-fit:
##
##   Fisher's C = 0 with P-value = 1 and on 0 degrees of freedom
##
## ---
## Coefficients:
##
##         Response          Predictor Estimate Std.Error DF Crit.Value P.Value
##        Inhib_STD       Cortisol_STD   0.0661      0.22 12     0.3006  0.7689
##        Inhib_STD           CORT_STD   0.2149    0.2152 12     0.9987  0.3376
##        Inhib_STD Body_condition_STD   0.1446    0.2129 12     0.6790  0.5100
##         otus_STD       Cortisol_STD   0.2150    0.2286 12     0.9406  0.3655
##         otus_STD           CORT_STD  -0.0209    0.2248 12    -0.0929  0.9275
##         otus_STD Body_condition_STD   0.1926    0.2311 12     0.8336  0.4208
##     Cortisol_STD Body_condition_STD  -0.0491    0.2136 14    -0.2297  0.8216
##         CORT_STD Body_condition_STD  -0.2430    0.2318 14    -1.0483  0.3123
##      ~~Inhib_STD         ~~otus_STD   0.0586         - 22     0.2558  0.4004
##   ~~Cortisol_STD         ~~CORT_STD   0.1298         - 22     0.5706  0.2875
##   Std.Estimate
##         0.0689
##         0.2336
##         0.1575
```

```
##         0.2131
##        -0.0216
##         0.1997
##        -0.0513
##        -0.2436
##         0.0586
##         0.1298
##
##   Signif. codes:  0 '***' 0.001 '**' 0.01 '*' 0.05
##
## ---
## Individual R-squared:
##
##       Response method Marginal Conditional
##      Inhib_STD   none     0.06        0.06
##       otus_STD   none     0.07        0.11
##   Cortisol_STD   none     0.00        0.00
##       CORT_STD   none     0.05        0.13
```

```
plot(path.mod2)
```

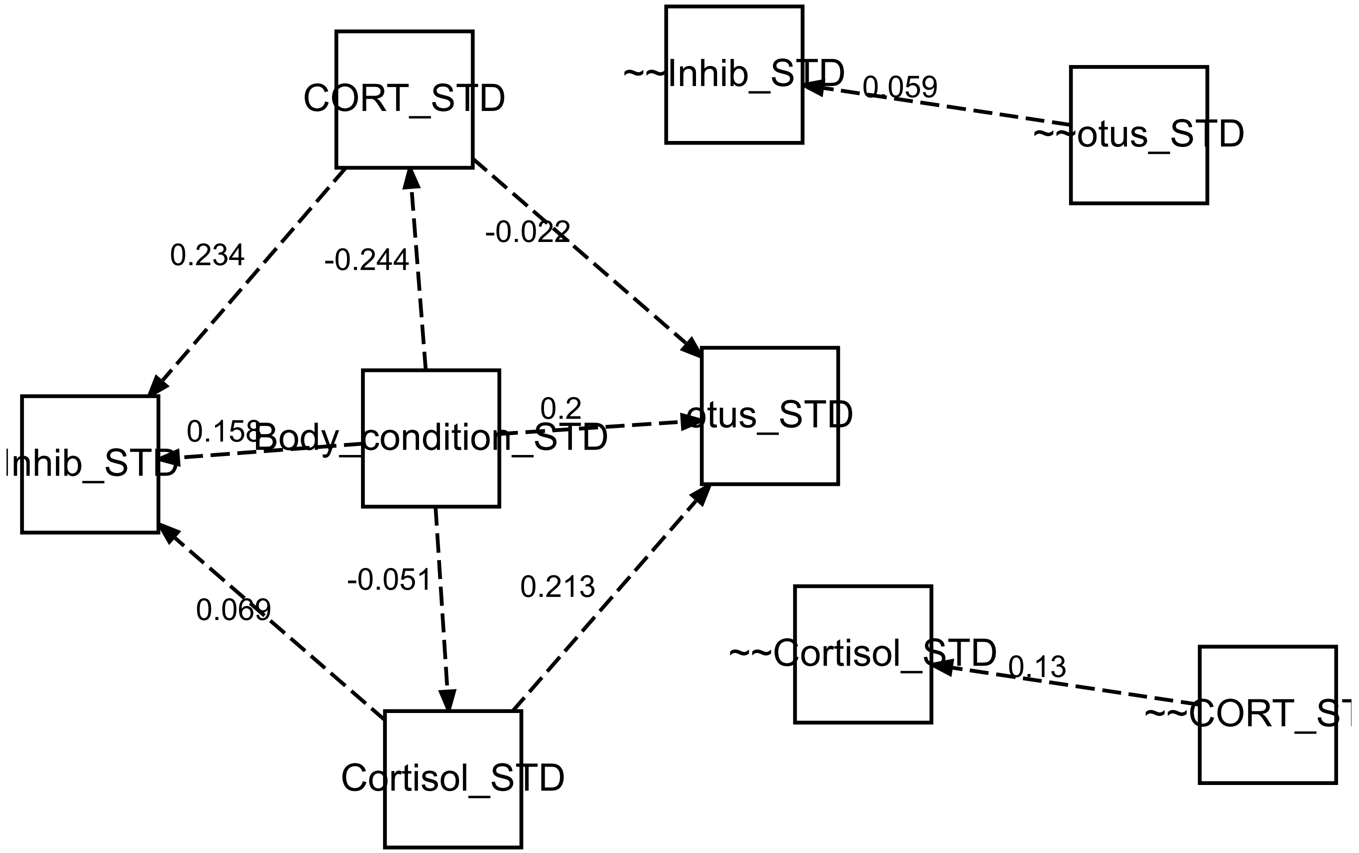
CORT_STD
~~Inhib_STD
0.059
~~otus_STD
0.234
-0.244
-0.022
0.158
Body_condition_STD
0.2
otus_STD
Inhib_STD
-0.051
0.213
0.069
~~Cortisol_STD
0.13
~~CORT_ST
Cortisol_STD

Fragment-Translocated

```
path.mod3 <- psem(
  mod1 <- lme(Inhib_STD ~ Cortisol_STD + CORT_STD + Body_condition_STD, random=~1|Frog_ID, correlation=corAR1(0, form = ~Days_Since_Release|Frog_ID), data=data_Frag[-c(4,25),]),
  mod2 <- lme(otus_STD ~ Cortisol_STD + CORT_STD + Body_condition_STD, random=~1|Frog_ID, correlation=corAR1(0, form = ~Days_Since_Release|Frog_ID), data=data_Frag[-c(4,25),]),
  mod3 <- lme(Cortisol_STD ~ Body_condition_STD, random=~1|Frog_ID, correlation=corAR1(0, form = ~Days_Since_Release|Frog_ID), data=data_Frag[-c(4,25),]),
    mod4 <- lme(CORT_STD ~ Body_condition_STD, random=~1|Frog_ID, correlation=corAR1(0, form = ~Days_Since_Release|Frog_ID), data=data_Frag[-c(4,25),]),
  Inhib_STD %~~% otus_STD,
  Cortisol_STD %~~% CORT_STD
)
summary(path.mod3)
```

```
##
## Structural Equation Model of path.mod3
##
## Call:
##   Inhib_STD ~ Cortisol_STD + CORT_STD + Body_condition_STD
##   otus_STD ~ Cortisol_STD + CORT_STD + Body_condition_STD
##   Cortisol_STD ~ Body_condition_STD
##   CORT_STD ~ Body_condition_STD
##   Inhib_STD ~~ otus_STD
##   Cortisol_STD ~~ CORT_STD
##
##     AIC      BIC
##  48.000   89.126
##
## ---
## Tests of directed separation:
##
##  No independence claims present. Tests of directed separation not possible.
##
## Global goodness-of-fit:
##
##   Fisher's C = 0 with P-value = 1 and on 0 degrees of freedom
##
## ---
## Coefficients:
##
##         Response          Predictor Estimate Std.Error DF Crit.Value P.Value
##        Inhib_STD       Cortisol_STD  -0.3453    0.1516 24    -2.2771  0.0320
##        Inhib_STD           CORT_STD   0.1409    0.1443 24     0.9761  0.3388
##        Inhib_STD Body_condition_STD  -0.1506    0.1533 24    -0.9822  0.3358
##         otus_STD       Cortisol_STD   0.0589    0.1597 24     0.3688  0.7155
##         otus_STD           CORT_STD  -0.4543    0.1537 24    -2.9547  0.0069
##         otus_STD Body_condition_STD   0.2764    0.1593 24     1.7348  0.0956
##     Cortisol_STD Body_condition_STD  -0.0135    0.1725 26    -0.0784  0.9381
##         CORT_STD Body_condition_STD  -0.1241     0.174 26    -0.7133  0.4820
##      ~~Inhib_STD         ~~otus_STD  -0.0924         - 41    -0.5721  0.2853
##   ~~Cortisol_STD         ~~CORT_STD   0.1945         - 41     1.2224  0.1145
##   Std.Estimate
##        -0.3572  *
##         0.1457
##        -0.1586
```

```
##        0.0579
##       -0.4464 **
##        0.2765
##       -0.0138
##       -0.1264
##       -0.0924
##        0.1945
##
##   Signif. codes:  0 '***' 0.001 '**' 0.01 '*' 0.05
##
## ---
## Individual R-squared:
##
##       Response method Marginal Conditional
##      Inhib_STD   none     0.14        0.31
##       otus_STD   none     0.24        0.35
##   Cortisol_STD   none     0.00        0.46
##       CORT_STD   none     0.01        0.22
```

```
plot(path.mod3)
```

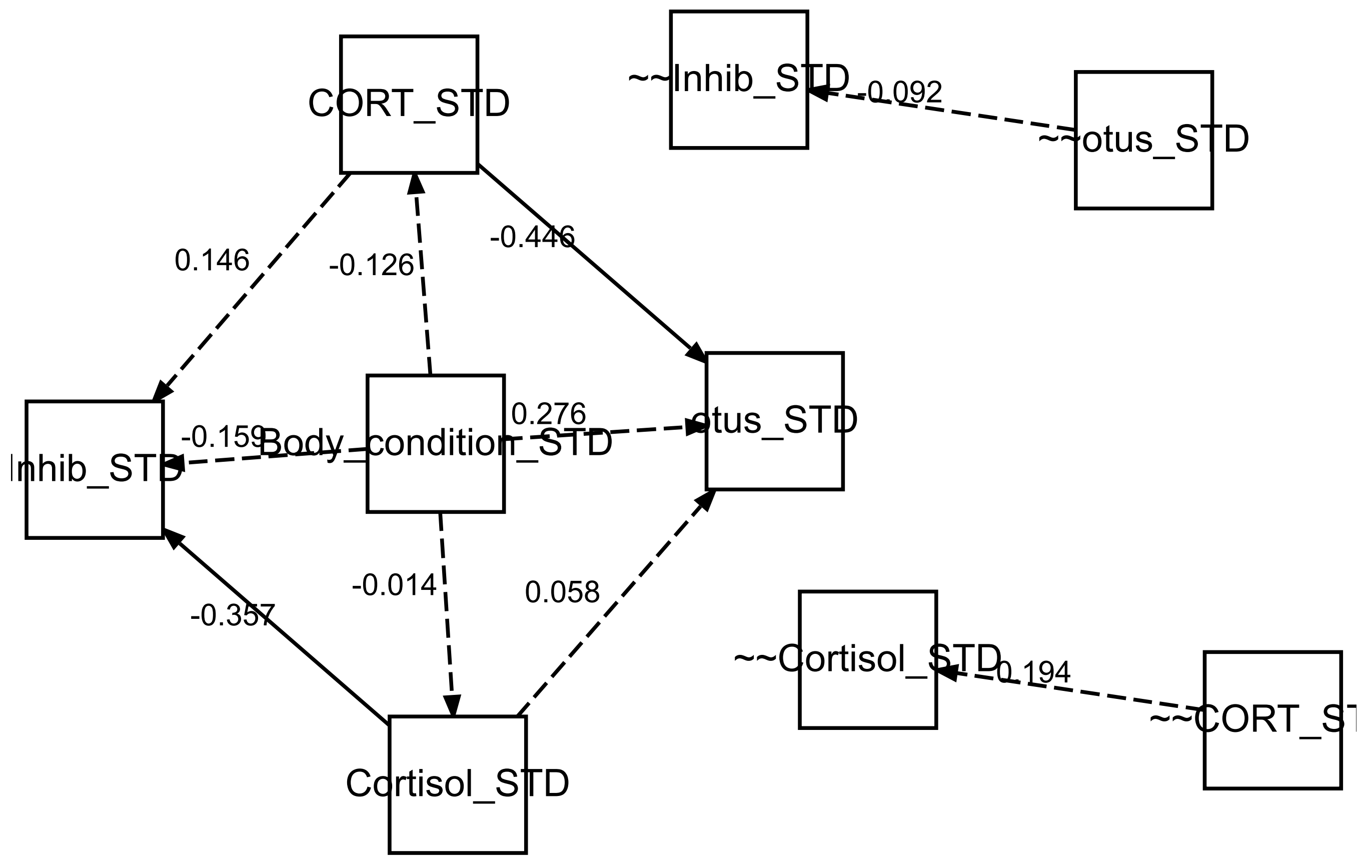
CORT_STD
~~Inhib_STD
-0.092
~~otus_STD
0.146
-0.126
-0.446
Inhib_STD
-0.159
Body_condition_STD
0.276
otus_STD
-0.014
0.058
-0.357
~~Cortisol_STD
0.194
~~CORT_ST
Cortisol_STD
