# Supplementary material for: Linking microbiome and stress hormone responses in wild tropical treefrogs across continuous and fragmented forests
Source: Commun Biol. 2023 Dec 12;6:1261. doi: 10.1038/s42003-023-05600-9 (PMC10716138; doi:10.1038/s42003-023-05600-9)
Supplement: Supplementary file 9 — Reporting Summary [file 42003_2023_5600_MOESM9_ESM.pdf]

nature portfolio

Corresponding author(s): Wesley J. Neely and C. Guilherme Becker

Last updated by author(s): Aug 4, 2023

Reporting Summary

Nature Portfolio wishes to improve the reproducibility of the work that we publish. This form provides structure for consistency and transparency in reporting. For further information on Nature Portfolio policies, see our Editorial Policies and the Editorial Policy Checklist.

Statistics

For all statistical analyses, confirm that the following items are present in the figure legend, table legend, main text, or Methods section.

n/a	Confirmed	
☐	☒	The exact sample size (*n*) for each experimental group/condition, given as a discrete number and unit of measurement
☐	☒	A statement on whether measurements were taken from distinct samples or whether the same sample was measured repeatedly
☐	☒	The statistical test(s) used AND whether they are one- or two-sided<br>*Only common tests should be described solely by name; describe more complex techniques in the Methods section.*
☐	☒	A description of all covariates tested
☐	☒	A description of any assumptions or corrections, such as tests of normality and adjustment for multiple comparisons
☐	☒	A full description of the statistical parameters including central tendency (e.g. means) or other basic estimates (e.g. regression coefficient) AND variation (e.g. standard deviation) or associated estimates of uncertainty (e.g. confidence intervals)
☐	☒	For null hypothesis testing, the test statistic (e.g. *F*, *t*, *r*) with confidence intervals, effect sizes, degrees of freedom and *P* value noted<br>*Give P values as exact values whenever suitable.*
☒	☐	For Bayesian analysis, information on the choice of priors and Markov chain Monte Carlo settings
☒	☐	For hierarchical and complex designs, identification of the appropriate level for tests and full reporting of outcomes
☒	☐	Estimates of effect sizes (e.g. Cohen's *d*, Pearson's *r*), indicating how they were calculated

Our web collection on statistics for biologists contains articles on many of the points above.

Software and code

Policy information about availability of computer code

Data collection	We used ArcGIS version 10.8.1 and Circuitscape version 4.0 to collect data on habitat resistance. We used QIIME2 to collect microbiome data from sequence reads.
Data analysis	We used R version 4.2.2, the open source piecewiseSEM package in R, the open source ecotraj package in R, the open source vegan package in R, the commercially available asreml package in R, Fragstats version 4.2.1, and JMP pro 15 for statistical analyses. Detail on analytical pipelines are given in the methods and supplemental files with R code created for this project are included with the manuscript.

For manuscripts utilizing custom algorithms or software that are central to the research but not yet described in published literature, software must be made available to editors and reviewers. We strongly encourage code deposition in a community repository (e.g. GitHub). See the Nature Portfolio guidelines for submitting code & software for further information.

Data

Policy information about availability of data

All manuscripts must include a data availability statement. This statement should provide the following information, where applicable:

- Accession codes, unique identifiers, or web links for publicly available datasets
- A description of any restrictions on data availability
- For clinical datasets or third party data, please ensure that the statement adheres to our policy

Raw data and R code used in analyses are available as supplemental files. All bacterial sequences are deposited in the NCBI Sequence Read Archive (BioProject PRJNA940133). Any additional data are available upon request.

Research involving human participants, their data, or biological material

Policy information about studies with human participants or human data. See also policy information about sex, gender (identity/presentation), and sexual orientation and race, ethnicity and racism.

Reporting on sex and gender	N/A
Reporting on race, ethnicity, or other socially relevant groupings	N/A
Population characteristics	N/A
Recruitment	N/A
Ethics oversight	N/A

Note that full information on the approval of the study protocol must also be provided in the manuscript.

Field-specific reporting

Please select the one below that is the best fit for your research. If you are not sure, read the appropriate sections before making your selection.

☐ Life sciences ☐ Behavioural & social sciences ☒ Ecological, evolutionary & environmental sciences

For a reference copy of the document with all sections, see nature.com/documents/nr-reporting-summary-flat.pdf

Ecological, evolutionary & environmental sciences study design

All studies must disclose on these points even when the disclosure is negative.

Study description	In this study we employ radio tracking on a cohort of 25 translocated and 5 non-translocated tree frogs to analyze the relationship between microbiome composition, proportion of anti-pathogen microbes, glucocorticoid levels, body condition, and Bd infection in the context of habitat fragmentation. We split 30 radio-tagged frogs into groups of 5 frogs that we released at 6 sites: 1 non-translocated group at the site of initial capture (Continuous-Control treatment), 2 translocated groups to other sites within the same continuous forest (Continuous-Translocated treatment) and 3 translocated groups to separate forest fragments (Fragment-Translocated treatment).
Research sample	We used a total of 40 radio-tagged individuals of the forest-associated blacksmith treefrog, Boana faber, from a single forested site at the Pro-Mata Center, located in Sao Francisco de Paula, Rio Grande do Sul, Brazil. This species is a large-bodied tropical tree-frog, large enough to ethically carry radio transmitters, and based on previous research tends to have high infection loads of our focal pathogen. Additionally, this species moves through aquatic, arboreal, and terrestrial habitats over relatively short timeframes, making it a good target for a study of host movement and deforestation.
Sampling strategy	We hand captured 30 individuals from a single large pond at the start of the project. We then attached coded nanotag VHF transmitters (NTQB-6-1 - Lotek Wireless INC; operating frequency 140-175Mhz) using microcapillary silicone tubes threaded with 28 gauge galvanized steel wire in a belt design. We monitored individual movement using a Biotracker VHF radio receiver and a 3-element folding Yagi antenna. We recaptured frogs using radiotelemetry every ~5 days over 6 weeks (December 2019 – January 2020). Sample sizes were chosen to maximize sample recovery at each site. Radio tracking was difficult in this mountainous environment, so 5 frogs was the maximum number able to be recaptured within a single day, when including driving to and from sites. With the repeated measures nature of these data, our sample sizes are sufficient for downstream analyses.
Data collection	At initial capture and each recapture, we collected precise GPS coordinates, measured snout-vent length (SVL) and body mass, and collected skin swabs from frogs following standard methods. Wesley Neely was involved in all sample collection, and collected all skin swabs used for pathogen detection, skin microbe analyses, and glucocorticoid quantification.

Timing and spatial scale	We recaptured frogs every ~5 days after initial release over 6 weeks (December 3rd 2019 – January 8th 2020). We visited sites sequentially, one sampled each day on rotation. This sampling procedure was done out of necessity, due to the long hours it takes to recapture and sample each frog. Additionally, landowner availability for property access was generally restricted to daytimes. Our work all occurred within 15 kilometers of our site of initial capture.
Data exclusions	Some samples were excluded due to rarefaction of microbial community data, or due to errors in glucocorticoid hormone quantification. For analyses, we removed samples with no recaptures and occasionally removed and sampled recaptures after the fourth. This exclusion criteria was necessary for comparability between treatments.
Reproducibility	We outfitted 5 frogs with radio transmitters at each site, for 5 replicates. We also replicated continuous forest sites and fragmented forest sites. The non-translocated site was not replicated since introducing variability from multiple sources of capture was determined to be a greater statistical weakness than lack of replication.
Randomization	Frogs were randomly allocated into groups of 5 that were then randomly allocated to a site of release.
Blinding	Blinding was not relevant to this study.

Did the study involve field work? ☒ Yes ☐ No

Field work, collection and transport

Field conditions	Our study site in southern Brazil was a high elevation sub-tropical rainforest characterized by consistently cool temperatures (15-20 C) and high humidity (75-85%). Rainfall was sporadic, but increased greatly towards the end of December.
Location	Our research was conducted in and around the Pró-Mata field station in São Francisco de Paula, Rio Grande do Sul, Brazil (-29.4809, -50.1752). This high elevation site is located at around 900 m above sea level and is characterized by mixed Araucaria deciduous forest and is flanked by large areas of grassland intermixed with forest patches and silviculture.
Access & import/export	We collaborated with the administrators at Pró-Mata field station to gain access to and lodging at all continuous forest. We received permission from all landowners prior to conducting research on their properties at forest fragments. All work was conducted in collaboration with a local university (Universidade do Vale do Rio dos Sinos) and under appropriate collection permits (Instituto Chico Mendes – SISBIO #70883-1) and institutional animal care and use approval (IACUC-UA #19-07-2547; CEUA-UNISINOS #PPECEUA10.2019).
Disturbance	Little to no disturbance was caused by this study. We kept sample sizes small in part for feasibility but also to minimize disturbance on frog populations during the breeding season.

Reporting for specific materials, systems and methods

We require information from authors about some types of materials, experimental systems and methods used in many studies. Here, indicate whether each material, system or method listed is relevant to your study. If you are not sure if a list item applies to your research, read the appropriate section before selecting a response.

Materials & experimental systems

n/a	Involved in the study	
☒	☐	Antibodies
☒	☐	Eukaryotic cell lines
☒	☐	Palaeontology and archaeology
☐	☒	Animals and other organisms
☒	☐	Clinical data
☒	☐	Dual use research of concern
☒	☐	Plants

Methods

n/a	Involved in the study	
☒	☐	ChIP-seq
☒	☐	Flow cytometry
☒	☐	MRI-based neuroimaging

Animals and other research organisms

Policy information about studies involving animals; ARRIVE guidelines recommended for reporting animal research, and Sex and Gender in Research

Laboratory animals	The study did not involve laboratory animals.
Wild animals	We hand captured 40 adult blacksmith treefrogs, Boana faber, from a single large breeding population. Frogs were kept in clean plastic bags filled with air until processing. Frogs were transported to translocation sites by car. After the study, frogs were released at the location of last capture after removing radio tags.

Reporting on sex	Sex was not considered in the study design, however mostly males were captured due to ease of capture when calling.
Field-collected samples	The study did not involve samples collected from the field.
Ethics oversight	All work was conducted under appropriate collection permits (Instituto Chico Mendes – SISBIO #70883-1) and institutional animal care and use approval (IACUC-UA #19-07-2547; CEUA-UNISINOS #PPECEUA10.2019).

Note that full information on the approval of the study protocol must also be provided in the manuscript.
